# Domain insertion permissibility-guided engineering of allostery in ion channels

**DOI:** 10.1038/s41467-018-08171-0

**Published:** 2019-01-17

**Authors:** Willow Coyote-Maestas, Yungui He, Chad L. Myers, Daniel Schmidt

**Affiliations:** 1 Department of Biochemistry, Molecular Biology & Biophysics, University of Minnesota, Minneapolis, 55455 MN USA; 2 Department of Genetics, Cell Biology & Development, University of Minnesota, Minneapolis, 55455 MN USA; 3 Department of Computer Science and Engineering, University of Minnesota, Minneapolis, 55455 MN USA

## Abstract

Allostery is a fundamental principle of protein regulation that remains hard to engineer, particularly in membrane proteins such as ion channels. Here we use human Inward Rectifier K^+^ Channel Kir2.1 to map site-specific permissibility to the insertion of domains with different biophysical properties. We find that permissibility is best explained by dynamic protein properties, such as conformational flexibility. Several regions in Kir2.1 that are equivalent to those regulated in homologs, such as G-protein-gated inward rectifier K^+^ channels (GIRK), have differential permissibility; that is, for these sites permissibility depends on the structural properties of the inserted domain. Our data and the well-established link between protein dynamics and allostery led us to propose that differential permissibility is a metric of latent allosteric capacity in Kir2.1. In support of this notion, inserting light-switchable domains into sites with predicted latent allosteric capacity renders Kir2.1 activity sensitive to light.

## Abstract (web summary)

Allostery is a fundamental principle of protein regulation that remains challenging to engineer. Here authors screen human Inward Rectifier K + Channel Kir2.1 for permissibility to domain insertions and propose that differential permissibility is a metric of latent allosteric capacity in Kir2.1.

## Introduction

Allostery is the phenomenon in proteins where the state of proximal sites is coupled to the state of distal sites. In nature, allosteric regulation is widespread in multidomain proteins, such as plant photoreceptors^1^, that arise from the recombination of functionally and structurally discrete protein domains. In the lab, we recombine domains to generate synthetic proteins; for example, antibodies that are joined end to end with signaling domains to create chimeric T-cell receptors for immunotherapy^2^. In both scenarios, how these components become allosterically coupled is essentially trial and error. That blind trial and error can progressively lead to optimized design through natural selection over billions of years is a central concept in the evolution of natural systems^3^. However, in the lab, we need to accomplish this task in less time and with greater efficiency.

One class of proteins that are challenging to engineer rationally are ion channels. Ion channels play critical roles in the biological signaling processes that determine the operation of cells and networks of the brain and the heart and are thus major drug targets^4^. Virtually every aspect of ion channel gating relies on allosteric regulation and many drugs achieve their therapeutic effect through allosteric modulation^5^. Being able to engineer the allosteric regulation of ion channels de novo, for example as chemo- or optogenetic tools^6,7^, would enable fine-tuned control and thus exploration of how individual channels contribute to cell physiology.

Models of allostery that could aid us in this task have continued to develop since the initial description of allostery as a phenomenon in proteins and the structure of hemoglobin, the prototypical allosteric protein^8^. Models in which allostery emerges from dynamic (entropic) mechanisms instead of structurally distinct macroscopic conformations have long been considered^10^. These models reconcile numerous phenomena such as instrinsically disorded proteins facilitating long range allosteric regulation^9^, that allostery can occur without structural change^10^, negative cooperativity^11^, and agonism/antagonism switching^12^. One framework, the ensemble allosteric model (EAM), unifies the classic Monod–Wyman–Changeux (MWC) and Koshland, Nemethy, Filmer (KNF) models with allostery emerging from intrinsic disorder and conformational fluctuations^13^. The EAM model describes allostery as thermodynamic interdependence of a protein’s cooperative structural elements, whose intrinsic stabilities are influenced by ligand binding^12,14^. The practical utility of the EAM models for engineering allostery was recently tested by guiding the engineering of a protein switch^15^. A defining feature of the EAM model is that allostery does not rely on specific obligatory allosteric pathways in proteins, but instead arises from the energetic balance of all structural elements within the protein—the conformational ensemble. In this view, a protein’s primary sequence not only encodes the tertiary structure, but also a protein’s energy landscape, which manifests as a protein’s conformational ensemble. Any perturbation that modulates the stability of a structural element will affect the stability of all other coupled elements. If allosteric coupling has such degenerate requirements, it is easy to see how a protein, in addition to its primary function, can possess latent functions that are not under selection^16,17^. These latent functions could become exploited and facilitated by the same amino acid sequence if selection pressures change. Latent function could be considered by-products (“spandrels”^18^) of the energy landscape topography. Latent phenotypes are co-opted in numerous biological contexts, including soluble proteins such as enzymes^19^ and hormone receptors^20^. As another example, a scaffold protein (Ste5) allosterically regulates Erk-like kinases that diverged before the evolution of Ste5 itself, implying that the allosteric capacity to be regulated was already present at that point^16^.

How is the notion of latent allostery relevant to ion channels? The majority (43 out of 45) of human ion channel families appeared in early metazoans^21^, so any subsequent functional diversification could conceivably be the result of leveraging latent regulatory mechanisms that existed in ancestral ion channel clades. However, it is unclear whether (1) ancestral channels used latent pathways to diversify function, (2) modern ion channels still possess latent allostery, and (3) whether latent allostery can be leveraged to engineer allosteric regulation into modern channels.

Approaching these questions from the perspective of the EAM model, we note that the greatest allosteric coupling response is observed when at least one of the involved structural elements is “poised” to undergo disorder/order transitions^14^. A structural element is poised when its intrinsic stability is such that it undergoes local disorder/order transitions. We therefore hypothesized that a good probe for allosteric capacity would be one that could determine regional conformational flexibility. We furthermore hypothesized that conformational flexibility would manifest as regional structural plasticity, which can be examined by measuring the permissibility of this region to a domain insertion. Regions that are conformationally flexible (fully or partially disordered) are more tolerant to an in-frame insertion of a protein motif (the probe) than regions that are well-structured (ordered). Conceptually, the idea of probing regional or site-specific structural plasticity, and more generally the idea of functionally linking together protein domains through domain insertion and recombination, is well accepted. It has been applied to the engineering of enzymes^22–24^, ion channels^25,26^, sensors for cellular states^27^, and ligand/metabolite sensors^28,29^. While approaches vary greatly, ranging from random^22,24,28^, evolution-based^30,31^, and structure-aided rational design^23^, domain insertion profiling with DNA sequencing (DIP-seq)^28,32^ is particularly well suited to probing latent allostery. DIP-Seq combines rapid insertion library generation with high-throughput assays that can link a functional fusion protein (phenotype) to the specific insertion product (genotype). Because DIP-Seq is unbiased and agnostic of the underlying mechanisms that give rise to allostery, it can broadly query a protein’s allosteric capacity.

In our view, the power of DIP-Seq for mapping allosteric capacity could be greatly improved if we explicitly examine how permissibility, for the same host protein, depends on the insertion of different domains (differential permissibility)—differential DIP-Seq. We argue this as follows: regions of a protein that are strongly biased toward order (e.g., transmembrane helices) are nonpermissive to any type of inserted domain because insertions will disrupt this region’s energetic balance and break the secondary and tertiary structure elements crucial for folding, trafficking, or multimeric assembly. Conversely, regions strongly biased toward disorder (e.g., unstructured termini) are generally permissive to any type of inserted domain because the enthalpic and entropic impact due to the domain insertion are minimal. An exception to this rule would be sites that contain trafficking and/other related signaling motifs^33,34^. Lastly, regions that are conformationally flexible and undergo disorder/order transitions; these regions are energetically balanced such that both ordered and disordered states are populated. In the limit that the perturbation introduced by insertion is relatively small compared to the free energy required to unfold this region, changes to the intrinsic stability of this region and consequently overall protein stability will depend on the properties of the inserted domain. The EAM model provides a link between these expectations and allostery. If the emergence of allosteric regulation requires regions that are conformationally flexible and poised to undergo disorder/order transitions, and if differential permissibility is a metric for poised conformational flexibility, then differential permissibility can be used to map allosteric capacity.

**Fig. 1:**
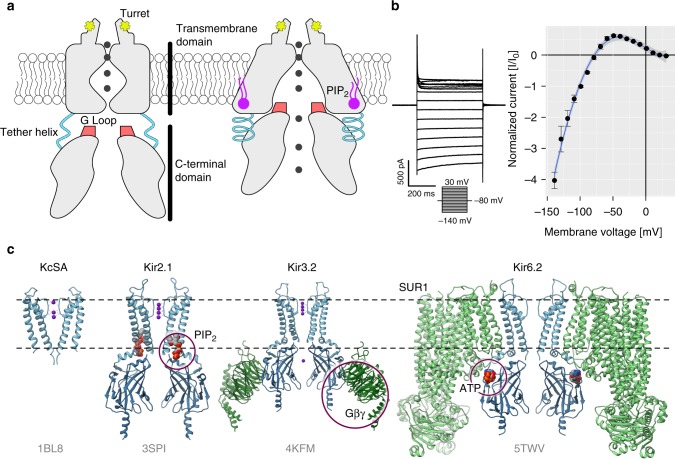
Inward rectifier K^+^ channels. **a** Domain architecture of Kir2.1 in the closed (left) and open conformations (right). The C-terminal domain (CTD) is connected to the transmembrane domain (TMD) via a tether helix (light blue). Upon binding of PIP_2_ (purple) at the interface between TMD and CTD, the tether helix undergoes a disorder-to-order transition and brings both domains closer together. The G-loop is wedged into the TMD causing the inner helix gate to open. Adapted from ref. ^37^. **b** Whole-cell patch clamp electrophysiology of WT Kir2.1 transiently expressed in HEK293FT cells. A representative recording (left) and normalized currents (right) show strong inward rectification ( ± s.e.m., *n* = 4). **c** A comparison between KcSA and representative structures of the inward rectifier K^+^ channel family (PDB accession codes are shown in gray) reveals that overall domain architecture is conserved. Channels are shown as blue and accessory protein as green ribbons. Allosteric modulators are indicated with purple circles

Here, we challenge these hypotheses and predictions and study exploited and latent allostery in inward rectifier K^+^ channel Kir2.1 via differential DIP-Seq. Inward rectifier K^+^ channels (Kir) are tetrameric K^+^ channels with diverse physiological roles^35^. They regulate resting membrane potential and excitability in neuromuscular tissue and vascular tone on blood vessels. They are involved in mechanisms of drug abuse and addiction, as well as learning by modulating synaptic plasticity. In the pancreas, they regulate insulin secretion. In recent years, several crystal and cryo-EM structures have improved our understanding of their gating mechanisms^36–39^. In Kir2.1 for example, the binding of the positive allosteric regulator PIP_2_ induces a disorder-to-order transition of a tether helix^37^. Because of that transition, the G-loop, located within the C-terminal domain (CTD), wedges into the transmembrane domain and forces the intracellular gate open allowing K^+^ to flow (Fig. 1a). Under physiological conditions, Kir channels generate an inward K^+^ current at potentials negative to reversal potential for K^+^ (Fig. 1b). They also permit some current at slightly more positive voltages before becoming blocked by Mg^2+^ and polyamines^40,41^. Interestingly, other members of the inward rectifier family share the overall topology (Fig. 1c), but are regulated by other ligands, including Gβγ (Kir3.x) and ATP (Kir6.x) binding in distinct regions of the CTD. Together, the availability of high-resolution structures^36–39^, functional studies (reviewed in ref. ^35^), and ability to express in heterologous systems, make Kir ideal test cases for applying the dDIP-Seq workflow.

We measure how permissive each ion channel site is to insertion of three different motifs with different physiochemical properties. We confirm that permissibility can be explained by conformational flexibility, and that differential permissibility is a hallmark of sites in Kir2.1 that are involved in allosteric regulation or that are corresponding to sites exploited for allosteric regulation in homologs of Kir2.1. We furthermore demonstrate that this framework of measuring differential permissibility in ion channels is useful to endow them with new functions. We discuss implications of differential permissibility with respect to rationalizing how function diversified during ion channel evolution.

## Results

### A high-throughput ion channel domain insertion pipeline

The EAM model^13^ predicts that regions with allosteric capacity (exploited or latent) are poised to undergo disorder/order transitions. Because inserting domains with different physiochemical properties will perturb the energetic balance in these regions to different degrees, we hypothesized that sites with allosteric capacity would have context-dependent mutability.

To test this idea, we used the DIPSeq^28,32^ workflow to insert four different motifs (PDZ, Cib81, GSAG_2×_, and GSAG_3×_) into nearly every amino acid position of Kir2.1—DIP-Seq relies on MuA transposase to insert an antibiotic cassette into random positions of a gene (i.e., all six reading frames) (Supplementary Fig. 1). Upon antibiotic selection of variants with insertions, we replaced this cassette with a motif of interest using restriction sites at transposon ends. The transposition mechanism^28,42^ furthermore dictated that all insertions are flanked by short linkers, Ala-Ser and Gly-Ser-Ala at the N- and C-terminus, respectively. We used the 10- kDa mouse α-syntrophin PDZ domain (PDB 2PDZ) because it is well-structured and has been used to study how inserting large domains with known structure and function disrupts recipient protein activity^43^. Cryptochrome-interacting basic-helix-loop-helix (Cib81) is a similarly sized 9-kDa plant protein domain that forms a two-component switchable system with its binding partner, CRY2, after blue-light illumination^44^. We included flexible 0.9-kDa GSAG_x2_ and 1.3-kDa GSAG_x3_ linkers to establish a permissibility baseline.

After transiently expressing four insertion libraries into HEK293FT along with EGFP as a transfection marker, we measured site-specific insertion permissibility. Permissibility is defined as the site-specific ability of Kir2.1 to accept an insertion without disrupting folding, homotetramer assembly, and trafficking to the cell surface. We leveraged the fact that for an inward rectifier to be expressed on the cell surface, it must fold, oligomerize, and surface traffic^33,45,46^. Therefore, only permissive insertion variants can be fluorescently labeled via a FLAG epitope tag we inserted into an extracellular loop of Kir2.1 (Ser116)^47^. In this way, we collected two cell populations by fluorescence-activated cell sorting (FACS) (Supplementary Fig. 2): channel variants that express but do not surface express (EGFP^high^/anti-FLAG^low^) and those that do surface express (EGFP^high^/anti-FLAG^high^). From both populations, we isolated and sequenced plasmid DNA and aligned reads with the DIPSeq alignment pipeline^28,32^. The complete workflow, including library generation, flow cytometry, and NGS was performed in triplicate. We calculate permissibility as site-specific enrichment between not surface-expressed (NSE) and surface-expressed (SE) insertion variants:

1 $${\boldsymbol{F}}\left( {{\boldsymbol{i}},{\boldsymbol{j}}} \right) = \frac{{{\boldsymbol{r}}_{{\boldsymbol{j}}_{{\boldsymbol{SE}}}}^{\boldsymbol{i}}}}{{{\boldsymbol{t}}_{{\boldsymbol{j}}_{{\boldsymbol{SE}}}}}} - \frac{{{\boldsymbol{r}}_{{\boldsymbol{j}}_{{\boldsymbol{NSE}}}}^{\boldsymbol{i}}}}{{{\boldsymbol{t}}_{{\boldsymbol{j}}_{{\boldsymbol{NSE}}}}}}$$

where *r* is the number of reads at amino acid position *i*, in the *j*th dataset divided by *t*, the total number of reads in the *j*th given sample.

**Fig. 2:**
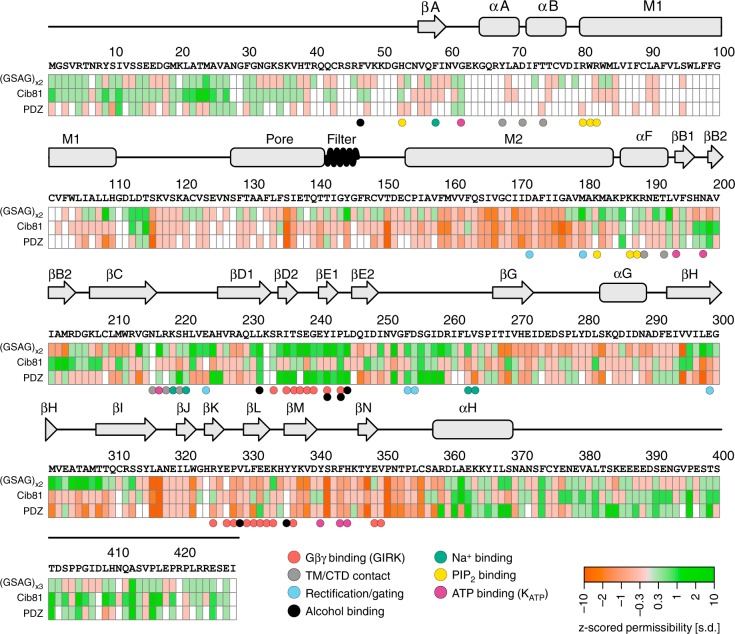
Domain insertion permissibility in hKir2.1. The primary sequence of human Kir2.1 (GI: 4504835) and secondary-structure elements are shown along with the permissibility score for three types of inserted domains (indicated on the left). Residues colored white refer to those for which there was insufficient data to assign a permissibility score. Key residues with functional relevance in Kir2.1 or a homolog (GIRK, K_ATP_) are indicated by color-coded spheres

Apart from some regions near the N-terminus, most notably M1, coverage in the remaining regions is near complete for all three insertion datasets (Fig. 2). While scanning mutagenesis^48^ of the M1 helix suggested that it likely would not allow domain insertions, the lack of data for the N-terminus, given its role in Kir2.1 gating^49^ and trafficking^34^, is unfortunate. That we consistently observed poor coverage in N-terminus can in part be explained by bias intrinsic to MuA transposases^50^.

### Permissibility is surprisingly different between domains

**Fig. 3:**
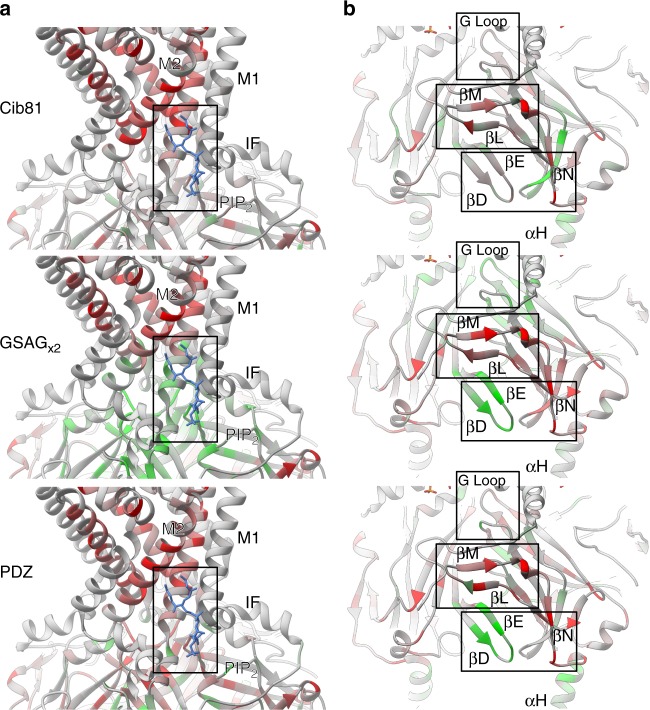
Differential domain insertion permissibility. Permissibility data is mapped on the crystal structure of chicken Kir2.2 (PDB 3SPI). Domain insertion permissibility for three different domains (indicated on the left) is shown colored increasing from red to green. **a** All types of insertions into transmembrane helixes (e.g., M2) are strongly selected against. Permissibility in PIP_2_ binding site (boxed) depends on structural context of the insertion. **b** Some non-conserved, surface-exposed loops (e.g., βN) were not permissive, while the βD–βE loop (which binds Gβγ in GIRK) and the G-loop (βH–βI, the cytoplasmic gate in Kir2.1) have context-dependent permissibility (i.e., permissive for GSAG_x2_ and PDZ insertion, less permissive for Cib81 insertion)

We then mapped permissibility onto the crystal structure of chicken Kir2.2 (PDB 3SPI)^37^. Kir2.1 and Kir2.2 are nearly identical apart from an extracellular loop between M1 and the pore helix. As expected, domain insertion positions that should not allow surface expression do not (e.g., transmembrane and inter-subunit interfaces, Fig. 3a, Supplementary Figs. 3–5). Unsurprisingly, the unstructured C-terminus (which in vivo interacts with scaffolding proteins not present in HEK293FT cells, such as PSD-95^51^) was highly permissive to any insertion (Fig. 2). Predictably, overall flexible peptide insertions are more permissive than larger, more structured domains. Counterintuitively, some surface-exposed, non-conserved regions (e.g., αG or βN, Kullback–Leibler divergence < 0.7, calculated for Pfam family IRK/PF01007 using MISTIC^52^) were also not permissive (Figs. 2, 3b). We take this as a data point suggesting that the permissibility rules, at least in Kir2.1, differ from those reported in other cytosolic proteins, such as kinases^23^. Perhaps, this is due to selection pressures unique to membrane proteins such as the need for proper folding, assembly, surface trafficking, and membrane insertion. Lastly, a surprisingly large fraction of Kir2.1 CTD was permissive to insertion of 10-kDa domains (5.4% of CTD residues, and 3.6% of Kir2.1 residues have a permissibility score of >2 standard deviations). Similar observations have been made in other proteins (e.g., refs. ^31,32^), and are thought to reflect that sequence continuity is not necessary for native folding^53^.

We more quantitatively compared permissibility profiles of biological replicates of Kir2.1 for inserted domains (structured vs. flexible) by clustering correlation matrices (Supplementary Fig. 6a). Biological replicates were overall in good agreement. This indicates that relatively little noise was introduced through the transposition, heterologous expression, and cell-sorting steps. We found that domains cluster by structure (Cib81 and PDZ cluster discretely from each other, but flexible insertions do not) and size (Cib81 and PDZ cluster closer than flexible peptides) (Supplementary Fig. 6a).

### Allosteric sites are most differentially permissive

Surprisingly, correlation matrices of permissibility profiles sorted by insertion sites reveal distinct clusters that coincide with structural features involved in allosteric regulation of Kir2.1 (Supplementary Fig. 6b). Permissibility in allosteric sites in Kir2.1 is more strongly correlated than in non-allosteric sites (unpaired Wilcoxon rank-sum test, *P*-value < 2.2 × 10^−16^). This included the PIP_2_ binding site at the interface between the pore and cytosolic domain, and the G-loop (βH–βI), a flexible region involved in channel opening^37^. The same pattern was observed in sites where allosteric modulators bind in homologs of Kir2.1. This includes the βB–βC loop (which binds ATP in Kir.6x^39^), and the βD–βE loop (which binds Gβγ in GIRK^38^).

Our data also revealed that permissibility is sensitive to the structural context of the inserted domain. Despite Cib81 and PDZ being of similar size, many sites were differentially permissive (69/229 sites, or 30%, when measured using the Hamming distance criterion after binarizing permissibility data). We interpreted this as context dependence for permissibility beyond simple steric effects (Figs. 2 and 3). While flexible linkers had the highest permissibility overall, in several sites, only Cib81 and PDZ are tolerated (e.g., βB2), further demonstrating permissibility’s context dependence. Furthermore, visual inspection of permissibility maps (Fig. 2, Supplementary Figs. 3–5) and a quantitative comparison of permissibility in PDZ and Cib81 datasets (using the Euclidean distance metric) shows that differential permissibility is a more common feature in functionally important regions (unpaired Wilcoxon rank-sum test, *P*-value = 3.3 × 10^−5^, Supplementary Fig. 7).

### Permissibility is dependent on dynamic protein properties

**Fig. 4:**
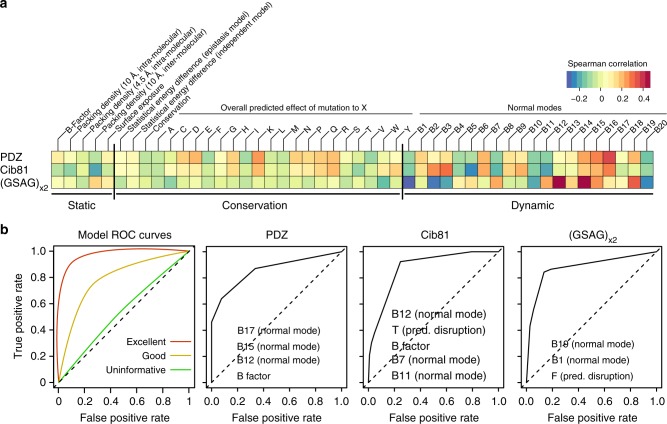
Parameter correlation and model performance. **a** Spearman correlations between permissibility for the inserted domain indicated on the left with the calculate property indicated on the top. Vertical bars separate feature categories (static, conservation, and dynamic). Dynamic protein properties show strong correlation, while correlation with static and conservation properties is spurious. **b** Model performance as measured with receiver operator characteristic (ROC) curves for each domain. Each decision tree model was evaluated using 10-fold cross-validation (see Methods for details). Example of ROC curves for models with varying performance are shown on the leftmost panel. For each domain, the top predictive properties used in decision trees are indicated on the ROC curve

Given that permissibility could not be explained by simple sterics, we explored what readily calculable and accessible protein features best explain permissibility, with the goal to derive a better understanding of the underlying mechanisms that determine permissibility. To this end, we calculated structure-, conservation, and dynamic-based properties for Kir2.1—using publicly available webservers (see Methods for more detail)—and calculated correlation coefficients between these properties and different domain permissibility profiles (Fig. 4a). We found that domain insertion permissibility is correlated with dynamic features and does not correlate well with static and conservation-related protein properties.

To further probe if correlation with dynamic features is meaningful (i.e., suggestive of mechanism) and determines how well computed properties explain permissibility, we constructed decision-tree classification models. Decision trees automatically pick features and thresholds based on experimental data to build a predictive model, which is then tested on withheld data. By observing which features get picked in the best performing models, we can deduce which protein properties predict and explain permissibility best. Consistent with the result that permissibility best correlates with dynamic properties, dynamic features had the greatest predictive power across the three types of domains (Fig. 4b, Supplementary Figs. 8–10). While some profiles’ predictive models perform better than others (GSAG_x2_ was best and PDZ was worse) and none of them fully explain permissibility, we can build models for all domains, whose performance is far better than random, as assessed by receiver-operating characteristic (ROC) curves and other performance criteria (Fig. 4b, Supplementary Fig. 10). Our ability to generate predictive models for permissibility demonstrates that features used in predicting measured permissibility are meaningful and that the indefinable qualities of permissibility play minor roles. That some properties, e.g., PDZ~B factors, Cib81~B12, and (GSAG)_x2_~F, are most predictive in decision-tree models yet are not strongly independently correlated demonstrates the superior sensitivity of nonlinear models that capture interactions between features (Fig. 4, Supplementary Fig. 8). Furthermore, the necessity for a nonlinear approach (decision trees) suggests that permissibility and by extension allosteric capacity, at least in Kir2.1, is an emergent phenotype from interactions between multiple protein properties, as opposed to a linear combination of, for example, conservation and surface exposure^23^.

### Allosteric site insertions contextually impact function

**Fig. 5:**
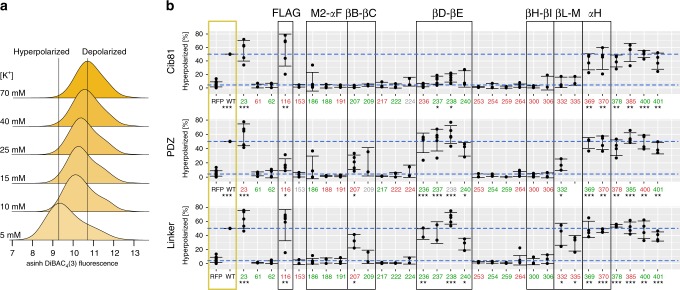
Domain insertion impact on Kir2.1 function. **a** Stacked histograms of population-level DiBAC_**4**_**(3)** fluorescence (hyperbole arcsine transformed) in HEK293FT cells expressing WT Kir2.1 as a function of external K^+^ (concentration indicated on the left). Increasing K^+^ depolarizes the cells, resulting in less membrane partitioning of the dye, thus increasing measured fluorescence. **b** The percentage of hyperpolarized cells expressing the indicated Kir2.1 variant and inserted domain are shown. Permissibility (Fig. 2) of that variant is indicated in color (green, permissive; red, nonpermissive; gray, no data). Higher percentage of hyperpolarized cells indicates function, and lower percentage of hyperpolarized cells indicates disruption. Reference measurements are provided for HEK239FT expressing miRFP670 alone and WT Kir2.1 co-expressed with miRFP670 (yellow box). Reference levels of WT and no channel are indicated by blue dashed lines. Replicates for insertion mutants are plotted with bars representing standard deviations and centered at the mean (each insertion variant *n* = 3–5, wild-type *n* = 21, and RFP670 *n* = 14). Significance of differences for means of each variant and WT with respect to channel (RFP) was tested using a one-sided t test. Significance levels are ***P < 0.001, **P < 0.01, and *P < 0.05, respectively. Variants without a mark are not significant. Regions discussed in the text are indicated by black boxes

Permissibility reports whether a Kir2.1 insertion variant can fold and traffic to the cell surface. We speculated that a significant fraction of insertion variants retain function in the presence of a large domain. To further explore Kir2.1’s differential sensitivity to domain insertion in allosteric regions, we focused on a representative sample of insertion positions—drawn from known allosteric sites in Kir2.1 and homologs, and sites with qualitatively different permissibility patterns—to assess whether they remain functional (i.e., able to conduct K^+^) upon domain insertion. We subjected this subset to a flow cytometry-based optical activity assay that measures population-level resting membrane potential (RMP) in HEK293FT cells using an oxonol voltage sensor, DiBAC_**4**_**(3)** (Fig. 5a)^54^. Since Kir2.1 drives the RMP toward the reversal potential of K^+^, cells expressing functional Kir2.1 are more hyperpolarized compared to empty (RFP-only) cells (Fig. 5b). By measuring RMP in many thousands of cells, we can bypass cell-to-cell variability that can make determination of RMP for transiently transfected cells by patch-clamp electrophysiology burdensome, particularly for poor-expressing insertion variants. In fact, even distributions of DiBAC_**4**_**(3)** fluorescence for WT Kir2.1 transfected cells are not uniform (Fig. 5a), indicating a continuum of more and less hyperpolarized cells.

Permissibility and impact on function differ in many sites. Some permissive sites do not produce functional protein, while other nonpermissive sites can produce functional protein. We expect this lack of correlation because permissibility solely tests for folding, assembly, and trafficking to the cell surface. A variant might traffic worse (thus appear nonpermissive) but ultimately be functional, while a well-trafficked variant can be functionally compromised. An insertion’s impact on function nevertheless follows a pattern similar to permissibility. As expected, insertions into flexible and highly permissive regions (e.g., C-terminal residues) of Kir2.1 have little impact on function. All insertions into regions critical for gating (e.g., the G-loop βH–βI) break channel function. The effect of insertions into the exposed extracellular loop (Ser116) is subtler. Here, both Cib81 and a flexible linker are well tolerated, while PDZ impairs function significantly. The emerging theme of differential impact on function continues in other regions of Kir2.1, including the PIP_2_ binding site (M2-αF). Here, Pro186 is permissive to all insertions, but partial function remains with large domain insertions (PDZ and Cib81), while a flexible linker completely breaks channel function. Conversely, permissibility and impact on function tracked quite well for insertions into the βD–βE loop. Here, both PDZ and flexible insertions allowed near-wild-type channel function, while Cib81 greatly reduced channel function. Overall, as with permissibility, the functional assay shows that domain insertions impact function in a context-dependent manner that cannot be explained with simple sterics.

### Allosteric site insertions enable control of channels

**Fig. 6:**
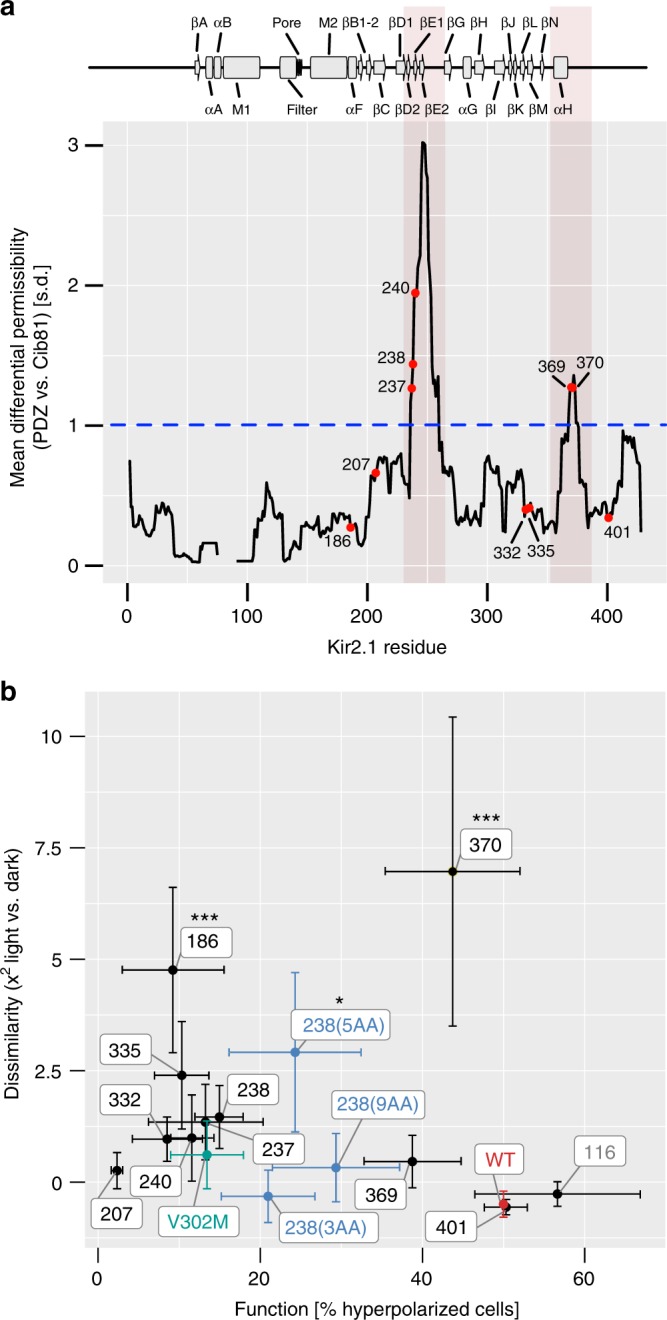
Light-modulated Kir2.1 variants. **a** Moving average (window size: 15 residues) of permissibility z-score difference between PDZ and Cib81 datasets. Regions in which differential permissibility exceeds one standard deviation (dashed blue line) are shaded in red. Individual residues subjected to the light-switching assay are shown as labeled red dots. **b** Dissimilarity (Χ^2^) of K^+^-induced depolarization with and without illumination (Supplementary Fig. 11) plotted against insertion variant function (Fig. 5). Mutants are highlighted after linker optimization (blue), gating mutant V302M (green), and wild-type Kir2.1 (red). Significance of light modulation is tested by Dunnett’s test for multiple comparisons with wild-type as control and post hoc multiple comparison adjustment. Error bars are standard deviations on the x-axis (each insertion variant *n* = 3–5, and wild-type *n* = 21) and s.e.m. on the *y*-axis (*n* = 3). Significance levels: ***P < 0.001; **P < 0.01; *P < 0.05, all others not significant

**Fig. 7:**
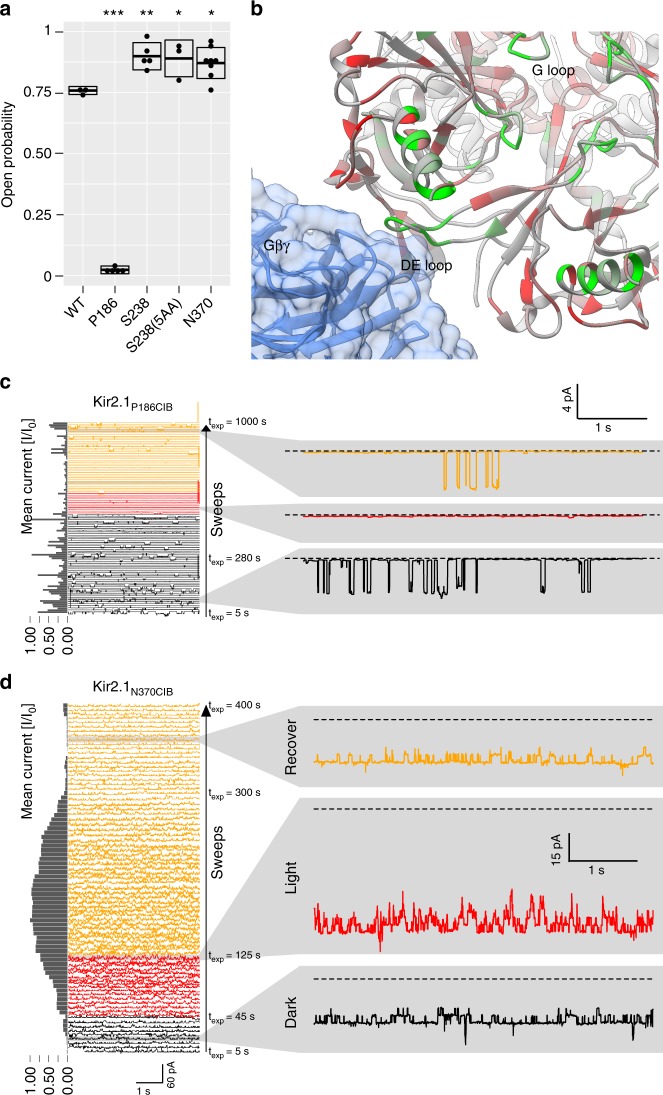
Electrophysiology of light-switched Kir2.1 variants. **a** Open probability determined by on-cell patch-clamp electrophysiology for WT Kir2.1 or the indicated insertion mutant. Boxes indicate standard deviation, thick crossbar indicates mean, and dots indicate individual measurements (*n* = 3–12). Significance is tested by Dunnett’s test for multiple comparisons with wild-type as control and post hoc multiple comparison adjustment. Significance levels: ***P < 0.001; **P < 0.01; *P < 0.05; all others not significant. **b** PDZ domain insertion permissibility mapped increasing from red to green onto the crystal structure of GIRK2 (Kir3.2) in complex Gβγ (PDB 4 KFM). Many highly permissive sites in Kir2.1 are homologous to those that interact with Gβγ in GIRK2. **c**, **d** Representative examples of on-cell patch-clamp recordings of indicated insertion variant (P186CIB top, N370CIB bottom). Consecutive 5-s sweeps that spread out vertically are shown, bottom (beginning of the experiment) to top (end of the experiment). Black indicates sweeps before illumination, red during illumination, and yellow after illumination. Holding voltage is −100 mV (P186CIB) and −80 mV (N370CIB). Channels open inward (negative current). Normalized mean current for each sweep is shown on the left as a bar graph. Magnified individual sweeps before (black), during (red), and after illumination (yellow) are shown on the right. While Kir2.1 P186CIB responds to illumination with further decreasing the already small open probability, Kir2.1N370CIB is activated with illumination

What permissibility assays tell us is that several sites in Kir2.1 appear to be sensitive to the structure of the inserted domain. Sites that are involved in allosteric signaling in Kir2.1 or are equivalent to functional sites in homologs (GIRK and Kir6.2) are more likely to be differentially permissive (Supplementary Fig. 7). Many of these sites retained partial function upon domain insertion (Fig. 5). In aggregate, we interpreted this data as sites with differential permissibility being more likely to possess allosteric capacity. We speculated that introducing light-switchable domains into allosteric sites might affect Kir2.1 with light and create an optogenetic reagent. To test this idea, we assayed Cib81 insertion variants for light-dependent modulation. Since it was not feasible to test all residues, we focused on those from allosterically regulated regions in Kir2.1 (M2-αF; Pro186) and in homologs such as Kir6.x (βB–βC; Lys207) and GIRK (βD–βE, βL–βM, αH; Thr237, Ser238, Glu241, Glu332, His335, Ser369, and Asn370). Controls included insertion into the unstructured C-terminus (Thr401), the extracellular loop Ser116, wild-type Kir2.1, and a pore-gating mutant, V302M^55^. Initially, there was no optimization of flanking linkers. We reasoned that if Cib81 was sterically accessible and the recipient site had allosteric capacity, then a light-mediated association of the channel with co-expressed Cry2 (size 70 kDa) would modulate channel gating even if binding interfaces are not optimized. We adopted the flow cytometry RMP assay to measure Kir2.1 activity after challenge with varying concentration of extracellular K^+^, with and without blue-light illumination. As expected, wild-type channel and a gating mutant had no light-dependent modulation (Fig. 6). Furthermore, when Cib81 was inserted into the extreme C-terminus (Thr401) or an inaccessible extracellular loop (Ser116), there was no light-dependent effect on Kir2.1 activity. Remarkably, even though channel function was severely impaired (Fig. 5b), when Cib81 was inserted in the PIP_2_ binding site, illumination markedly decreased the remaining Kir2.1 activity (Fig. 6b). We validated this with patch-clamp electrophysiology, which shows that the open probability of Kir2.1(Pro186CIB), which was low to begin with (Fig. 7a), was further decreased with blue-light illumination (Fig. 7c).

We also observed Kir2.1 light modulation when Cib81 was inserted into the pore-facing side of the αH helix (N370), but not the outward-facing side (Ser369) (Fig. 6b). Furthermore, patch-clamp validation of this insertion showed that open probability was higher than the wild-type channel in the absence of illumination (Fig. 7a) and further increased with illumination (Fig. 7d). The αH helix is a potential Gα-binding site in GIRK based on several NMR structures; however, this interaction has not been fully explored^56^.

We noticed weak light modulation when Cib81 was inserted into the βD–βE and βL–βM loops (e.g., Ser238, Leu332, and His335) (Fig. 6b). The weak impact of Cry2 recruitment in this region could be due to none-optimized binding interfaces in contrast to those Gβγ encounters in GIRK’s βD–βE and βL–βM loops (Fig. 7b)^38^. When we patched cells expressing these insertion mutants, we observed higher Kir2.1 activity in one (Ser238) even in the absence of illumination (Fig. 7a), suggesting that insertions into the βD–βE loop are activating. No channel activity was observed for Leu332 and His335. We explored linker optimization for a cleaner photoswitching phenotype. Flanking Cib81 by five amino acids, but not three or nine, improved light modulation of Ser238CIB (Fig. 6b).

## Discussion

Our findings reveal that permissibility in Kir2.1 is correlated with protein dynamics, but not structural features or conservation. There is broad support for the idea that the dynamics of structural elements in proteins, when poised, provide the mechanistic basis for allosteric coupling^13,14^. Protein dynamics are influenced by the stabilities of participating structural elements, which can be altered by in-frame domain insertions. The magnitude and sign (i.e., more ordered vs. more disordered) depends on the physicochemical properties of the inserted domain. This context dependence of altered local dynamics manifests in our assays as differential permissibility.

Translated into categories of permissibility, we expect generally permissive regions, where insertions are accepted and the overall functional phenotype of the channels remains mostly unchanged. Structurally important regions are expected to cause the misfolding and a loss-of-function phenotype regardless of the inserted domain’s properties. Regions that have conformational plasticity that depends on the context of a perturbation are expected to have differential permissibility. This means that the effect of a domain insertion on both folding, assembly, and trafficking will depend on the biophysical properties of the inserted domain. Similarly, different functional phenotypes are expected dependent on the context of the inserted domain.

We have found regions belonging to each of these three categories in Kir.2.1. Examples of the first category, generally permissive regions, are many sites toward the C-terminus, which have high permissibility to any domain insertion and where the impact on function is minimal, irrespective of insertion type. Universal permissibility likely means that these regions play minor roles in surface trafficking, oligomerization, or channel gating. This is consistent with their known role for interacting with binding partners, many of which are not present in HEK293FT cells^51^.

Examples for the second category, scaffold sites, include transmembrane helices, which have low permissibility to any insertion, and where any domain insertion severely impacts function. The universal disruptiveness of mutations within these regions is likely due to these regions being essential for folding, oligomerization, surface trafficking, or membrane insertion. This is consistent with many scaffold sites occurring within transmembrane domains or at interfaces between channel monomers.

Sites in the third category have differential permissibility that depends on both the structural context of the insertion position as well as the biophysical properties of the inserted domain. We postulate that these sites have latent allosteric capacity. Indeed, significant (> 1 standard deviation) differential permissibility was measured in the βD–βE loop and the αH helix (Fig. 6a). While the βD–βE loop has no known endogenous regulatory function in Kir2.1, it is part of an alcohol-binding pocket conserved in both Kir2.1 and GIRK^45,57^. Functional analysis in GIRK revealed that alcohol modulates PIP_2_-mediated channel activation in a G-protein-independent way^57,58^. Furthermore, the βD–βE loop is critical for mediating activating interactions between Gβγ and GIRK (Fig. 7b)^38^. That inserting PDZ or Cib81 domains, whose potential interaction surface is roughly similar to that of Gβγ’s, into this loop resulted in an activating phenotype suggests that Gβγ modulation of GIRK can perhaps be thought of as two different processes: one process is specific binding mediated by residues that exist in GIRK but not in Kir2.1, and the second process being a mechanism for coupling this binding to channel opening that exists both in GIRK—and—to a lesser extent—in Kir2.1, because of the shared architecture of the C-terminal domain. This type of division of labor, where one set of sites encodes affinity, while the other set encodes a filter for efficacy has been described for several types of allosteric regulation, including Gα activation of GPCR^59^ and ligand binding to bioamine receptors^60^.

Our data also suggest that determining differentially permissive sites, as opposed to simply permissive sites^32^, is useful to predict engineerable allosteric capacity. Inserting light-switchable domains into regions with significant differential permissibility rendered Kir2.1 activity sensitive to light (2 out of 2 regions; 2 out of 5 sites after linker optimization). Outside of the PIP_2_ binding site—where Cib81 insertions resulted in almost nonfunctional channels—we did not observe this in regions in which permissibility was less sensitive to context of the inserted domain (0 out of 4 sites), nor in other controls (0 out of 3 sites/controls). Our interpretation of this result is that mapping differential permissibility might represent a generalizable method to inform the de novo engineering of allosteric regulation in any protein. Further experiments are needed, of course, to fully test this idea. For example, by using permissibility mapping to render several endogenous ion channels sensitive to bio-orthogonal stimuli, such as drugs and light. Such tools have great utility for understanding how ion channels sculpt the function and adaptation of neuromuscular tissues, in both normal and pathological contexts. To this end, our ability to build predictive models of permissibility also means that if models are trained on a sufficiently large experimental dataset, it might be possible to derive generalized predictive models that can predict permissibility for potentially any ion channel, thus rendering case-by-case mapping of permissibility superfluous.

More specifically, it will be interesting to see if permissibility can be predicted from the same set of calculated properties for any ion channel (indicating permissibility is universal), or whether it is a function of phylogenetic distance (indicating permissibility is path dependent). Interpreted broadly, mapping and building models of permissibility—and by extension allostery—as it changes through phylogeny may be useful in explaining how specific ion channel families evolved. Much of the core functionality and architecture of ion channel families had evolved by the time the metazoan lineage appeared^21^. Subsequent diversification, driven by adaptive pressure to develop specialized neuromuscular tissue, can be considered fine-tuning biophysical properties and evolving regulation. We can observe this in K^+^ channels (Fig. 1c). After the inward rectifier architecture (represented by Kir2.1) evolved from the simpler pore-only architecture (KcSA) by the addition of the CTD, the same overall architecture is utilized for different modes of allosteric regulation, including different small-molecule ligands (PIP_2_, ATP, and Na^+^) and proteins (Gβγ and SUR1). The notion of latent allosteric capacity can explain how this came to be. Dynamic features for the most part arose from global architecture fine-tuned by local interactions^61^. Allosteric capacity is an emergent by-product of these dynamics features^17^. It is likely that allosteric regulation schemes leveraged pre-existing intrinsic properties of a protein’s structural elements, since this is the path that requires the fewest mutations to implement this function to come under selection.

## Methods

### MuA domain insertion library generation

Transposition libraries were generated using 100 ng of MuA-BsaI-engineered transposon and 1:2 molar ratio of transposition target DNA in 20-µl reactions with 4 µl of 5x MuA reaction buffer and 1 µl of 0.22 µg/µl MuA transposase (Thermo Fisher). MuA-BsaI-engineered transposon propagation plasmid or pUCKanR-Mu-BsaI was a gift from David Savage (Addgene plasmid # 79769)^28^. MuA-BsaI-engineered transposon was digested with BglII and HindIII Fastdigest enzymes (Thermo Fisher) and gel purified (Zymo Research).

The transposition target, human Kir2.1 (GI: 4504835, https://www.ncbi.nlm.nih.gov/protein/NP_000882), including a porcine teschovirus ribosomal skipping sequence (P2A)^62^, was codon-optimized for mouse, synthesized (Gen9), and subcloned with primers 1 and 2 (Supplementary Table 1) into pATT-Dest using NEB BamHI and HindIII. pATT-Dest was a gift from David Savage (Addgene plasmid # 79770)^28^. A FLAG tag was inserted before Ser116 using Q5 site-directed mutagenesis using primers 11 and 12 (Supplementary Table 1). MuA transposition reactions were incubated at 30 °C for 18 h for transposition, followed by 75°C for 10 min for heat inhibition. DNA from reactions was cleaned up (Zymo Research) and eluted in 10 µl of water. All 10 µl were transformed into 30 µl of electrocompetent 10G ELITE *E. coli* (Lucigen) in 1.0-mm Biorad cuvettes using a Bio-Rad Gene Pulser II electroporator (settings: 10 µF, 600 Ω, 1.8 kV). Cells were rescued and grown without antibiotics for 1 h at 37 °C. Aliquots were then serially diluted and plated on LB agar plates containing carbenicillin (100 µg/ml) and chloramphenicol (25 µg/ml) to assess library coverage. The remaining transformation mix was grown in 50 ml of LB containing carbenicillin (100 µg/ml) and chloramphenicol (25 µg/ml). All transformed libraries yielded greater than 10^5^ colonies, which for Kir2.1-P2A (1369 bp) is > 35x coverage. Plasmid DNA was purified by midi-prep kit (Zymo Research).

Transposition-inserted Kir2.1 variants were subcloned into an expression vector by amplifying channel variant genes using primers 3–6 (Supplementary Table 1) adding on BsmbI sites, using 10 cycles of PCR using Primestar GXL (Takara Clontech) and run on a 1% agarose gel. The larger band was cut out and gel purified (Zymo Research) to isolate channels with inserted transposons. A mammalian expression vector (pcDNA3.1) with EGFP was amplified to add on BsmbI sites complementary to those on Kir2.1-P2A using primers 7–10 (Supplementary Table 1). The Kir2.1-P2A (BsaI-transposon) variants were subcloned into this vector by BsmbI-mediated Golden Gate cloning^63^. Reactions were cleaned (Zymo Research) and eluted with 10 µl of water. All 10 µl were transformed into 30 µl of Lucigen electrocompetent 10G ELITE *E. coli* and electroporated in 1.0-mm Biorad cuvettes using a Bio-Rad Gene Pulser II electroporator (settings: 10 µF, 600 Ω, 1.8 kV). Cells were rescued and grown without antibiotics for 1 h at 37 °C and then with an aliquot serially diluted and plated on LB agar plates containing kanamycin (50 µg/ml) and chloramphenicol (25 µg/ml) to assess library coverage. The remaining transformation mix was grown in LB containing kanamycin (50 µg/ml) and chloramphenicol (25 µg/ml). All transformed libraries yielded greater than 10^5^ colonies, so for Kir2.1 (1369 bp) there is > 35x coverage. Plasmid DNA was purified by midi-prep kit (Zymo Research).

Inserted transposons were replaced with domains in individual reactions using BsaI-mediated Golden Gate cloning^63^. Domains (PDZ (GI: 404931, https://www.ncbi.nlm.nih.gov/protein/404931), Cib81^44^, GSAG_x2_, and GSAG_x3_) for insertions were ordered as gBlocks (IDT DNA), and PCR amplified to add on BsaI and linkers (Ala-Ser and Ser-Ala-Gly using primers 77–84 (Supplementary Table 1), preceding and following the domain insertion), sites complementary to MuA-BsaI-transposon sites for Golden Gate cloning. Domain amplicons were gel purified (Zymo Research). The product was further digested with AgeI-HF (NEB) and Plasmid-Safe ATP-dependent DNase (Epicentre) to remove any undigested transposon, then cleaned up (Zymo Research), and eluted with 10 µl of water. All 10 µl were transformed into 30 µl of Lucigen electrocompetent 10G ELITE *E. coli* and electroporated in 1.0-mm Biorad cuvettes using a Bio-Rad Gene Pulser II electroporator (settings: 10 µF, 600 Ω, 1.8 kV). Cells were rescued and grown without antibiotics for 1 h at 37 °C. An aliquot was serially diluted and plated on LB agar plates containing kanamycin (50 µg/ml) to assess library coverage. The remaining transformation mix was grown in LB containing kanamycin (50 µg/ml). All transformed libraries yielded greater than 10^5^ colonies, so for Kir2.1 (1369 bp) there is >35x coverage. Plasmid DNA was purified by midi-prep kit (Zymo Research).

### Domain insertion permissibility cell-sorting assay

 100 ng of each domain insertion library was transfected with 36 µl of turbofect (Thermo Fisher) into 50% confluent HEK293FT (Invitrogen, R70007) with 11.9 µg of dummy plasmid (pATT Dest) divided across a single six-well dish (9.6 cm^2^/well).

Cells from each well were detached using 1 ml of accutase (Stemcell Technologies) and twice spun down at 450×g and resuspended in FACS buffer (2% FBS, 0.1% NaN3, and 1xPBS). Cells were incubated with 1:200 anti-flag mouse antibody (Sigma, F1804) for 1 h rocking at 4 °C, washed twice with FACS buffer, covered with aluminum foil, and then incubated with 1:400 anti-mouse Alexa Fluorophore 568 (Thermo Fisher, A-11004) for 30 min rocking at 4 °C. Cells were washed twice, resuspended in 3 ml of FACS buffers, and filtered using cell strainer 5-ml tubes (Falcon). Cells were kept on ice and protected from light in the transfer to the flow cytometry core. Before cell sorting, a small aliquot of cells was saved as a control sample for sequencing.

Cells were sorted into EGFP^high^/Alexa Fluorophore 568^low^ (transfected cells without surface expression) and EGFP^high^/Alexa Fluorophore 568^high^ (transfected cells with surface expression) on a BD FACSAria II P69500132 flow cytometer. EGFP fluorescence was excited using a 488-nm laser, recorded with a 525/50-nm band-pass filter, and a 505-nm long-pass filter. Alexa fluorophore 568 fluorescence was excited using a 561-nm laser and recorded with a 610/20-nm band-pass filter. Cells were gated on side-scattering and forward-scattering areas to separate out whole HEK293FT cells, gated on forward-scattering height and width to separate single cells, then gated on co-expressed EGFP to gate out cells that received a plasmid, and then gated on cells that were labeled using the anti-flag antibody for surface-expressed channels. Gates were determined using a single wild-type, EGFP only, and unstained library samples. A representative example of this gating scheme is shown in Supplementary Fig. 12. EGFP^high^/label^low^ and EGFP^high^/label^high^ cells were collected into catch buffer (20% FBS, 0.1% NaN_3_, and 1xPBS). Between 2,000 and 100,000 cells were collected for each sample/library pair which is ~4–250x coverage of all potentially productive (i.e., in-frame and forward) domain insertions.

DNA from control, EGFP^high^/label^low^, and EGFP^high^/label^high^ cells for each library was extracted using a Microprep DNA kit (Zymo Research) and triple eluted with water. To remove chromosomal DNA, samples were digested with Plasmid-Safe ATP-dependent DNase (Epicentre). The resulting plasmid DNA was further purified and concentrated using midi-prep kit (Zymo Research). The product was used as a template for 12 cycles of PCR with primers 75–76 (Supplementary Table 1) using Primestar GXL (Takara Clontech), run on a 1% agarose gel, and gel purified (Zymo Research) to remove any primer dimers or none of the amplicon DNA. Purified DNA was quantified using Picogreen. Equal amounts of each domain insertion sample were pooled by cell-sorting category (control, EGFP^high^/label^low^, and EGFP^high^/label^high^ were pooled for library generation and sequencing).

### Sequencing

Libraries were generated at the University of Minnesota Genomics Core using Nextera XT or Nano Truseq library generation (Illumina) to fragment and add on Illumina sequencing adaptors and sequenced using either HiSeq or MiSeq sequencing platforms.

### Domain insertion permissibility alignment and enrichment

Alignments were done on both forward and reverse reads using a DIP-Seq pipeline^32^ developed by David Savage and coworkers that we slightly modified for compatibility with updated Python packages. Reads with duplicate domain insertion calls in both forward and reverse reads were removed. This pipeline produces plaintext files indicating a domain insertion position and whether that insertion is in-frame and in the forward direction. Enrichment was calculated by comparing the change in EGFP^high^/label^low^ to EGFP^high^/label^high^ cells. Only positions with reads in both samples were used in enrichment calculations. All other positions are treated as “NA” and not considered in downstream analysis and structure mappings, with the exception of calculating correlations between datasets and correlations between sites. In these correlation calculations, treat NA’s as 0’s, so removing all the data will introduce more noise when comparing between datasets due to limits from sampling.

Permissibility function for individual datasets comparing surface-expressed (SE) and non-surface-expressed (NSE) insertion variants:

2 $${\boldsymbol{F}}\left( {{\boldsymbol{i}},{\boldsymbol{j}}} \right) = \frac{{{\boldsymbol{r}}_{{\boldsymbol{j}}_{{\boldsymbol{SE}}}}^{\boldsymbol{i}}}}{{{\boldsymbol{t}}_{{\boldsymbol{j}}_{{\boldsymbol{SE}}}}}} - \frac{{{\boldsymbol{r}}_{{\boldsymbol{j}}_{{\boldsymbol{NSE}}}}^{\boldsymbol{i}}}}{{{\boldsymbol{t}}_{{\boldsymbol{j}}_{{\boldsymbol{NSE}}}}}}$$

where *r* is the number of reads at amino acid position *i*, in the *j*th dataset divided by *t*, the total number of reads in the *j*th given sample. The resulting data from individual sequencing reads are only used to calculate correlations between domains and amino acid positions.

For structure mappings and predictive model training, means of permissibility for a given domain insertion variant are used. So, the resulting mean permissibility function is

3 $${\boldsymbol{G}}\left( {{\boldsymbol{i}},{\boldsymbol{j}}} \right) =\frac{{{\sum} \left({\frac{{{\boldsymbol{r}}_{{\boldsymbol{j}}_{{\boldsymbol{SE}}}}^{\boldsymbol{i}}}}{{{\boldsymbol{t}}_{{\boldsymbol{j}}_{{\boldsymbol{SE}}}}}} - \frac{{{\boldsymbol{r}}_{{\boldsymbol{j}}_{{\boldsymbol{NSE}}}}^{\boldsymbol{i}}}}{{{\boldsymbol{t}}_{{\boldsymbol{j}}_{{\boldsymbol{NSE}}}}}}} \right)}}{{\boldsymbol{n}}}$$

where *r* is the number of reads at amino acid position *i*, in the *j*th dataset divided by *t*, the total number of reads in that given sample, summed for all replicates of that domain-channel combination, and divided by *n*, the number of datasets.

Mean permissibility was z-scored and mapped onto the structure of chicken Kir2.2 (PDB 3SPI) using Chimera^64^. Mapped dataset for Cib81, PDZ, and GSAG_x2_ linker had adequate coverage: 76.6% Cib81 (333/435), 68.9% PDZ (300/435), and 76.7% (334/435) of potential amino acid positions.

### Dataset correlations

Pearson correlations were used to calculate correlations between domain insertion datasets. Pearson correlations were also used to calculate correlations between amino acid positions across all datasets. Both these correlation matrices were calculated using the dataset that was trimmed to avoid sampling problems such that no more than 0.375 (6/16) datasets are where raw fitness was –1×10^–4^ < x < +1×10^–4^. These correlations were calculated with 63% of possible positions (277/435).

Spearman correlations were used to compare mean domain insertion datasets and calculated protein properties because Spearman correlations are often better at handling nonparametric correlations. Based on lack of structural and conservational data at various amino acid positions, many sites had to be trimmed. Data were trimmed from positions where more than half of the datasets had a raw mean fitness that was –1×10^–4^< x < +1×10^–4^. This resulted in datasets that contained 70% of possible positions (207/293).

### Computed protein properties

Static protein properties (B-factor, 10 Å intra-monomeric packing density, 4.5 Å intra-monomeric packing density, 4.5 Å inter-monomeric packing density, and surface exposure) were calculated using the SWIFT web server^65^ on chicken Kir2.2 (PDB: 3SPI)^37^, conservation-based properties (overall predicted disruptive effect of a mutation, conservation, and individual predicted disruptive effect of a mutation to specific amino acids (A, C, D, E, F, G, H, I, K, L, M, P, Q, R, S, T, V, W, Y)) were acquired from the EVmutation data server^66^, and dynamic properties (first 20 normal modes of the B monomer) were calculated on the iGNM 2.0 normal mode web server^67^. After trimming, computed protein properties were calculated for all parameters for 67% (293/435) of possible positions.

### Decision-tree models

We chose decision trees for building predictive models due to their utility in handling and determining nonlinear interactions. Prior to training models, data were binarized such that 0 was not permissive and 1 permissive for a given domain insertion. After trimming any data for a given mean raw fitness between –1×10^–4^ < x < +1×10^–4^ datasets, models were trained on 62.5% (183/293) Cib81, 58.0% (170/293) PDZ, and 68.6% (201/293) of possible positions. Models were limited to a depth of 4 to minimize model overfitting, trained on the computed protein properties to predict Cib81, PDZ, and GSAG_x2_ using the rpart package^68^ in R, and cross-validated 10 times. Model performance was determined using commonly used criteria: receiver- operating characteristic (ROC) curves, the complexity parameter (used in minimizing tree size)/tree depth and model residuals, precision vs. recall, accuracy vs. cutoff, precision vs. cutoff, and recall vs. cutoff (Supplementary Fig. 10). As further validation, models were trained as only the four most significant protein properties based on Spearman correlations to demonstrate necessity and utility of using decision trees vs. correlation calculations (Supplementary Fig. 9A), by withholding the most important properties determined with decision tree and by withholding whole classes of protein properties (static, conservation, and dynamic, Supplementary Fig. 9B).

### Resting membrane potential functional assay

Single mutants were generated by inserting a BsaI site and a five-basepair replication identical to those created by transposons that replicated the beginnings and ends of transposon-mediated domain insertions using a Q5 site-directed mutagenesis kit (NEB) with primers 13–72 (Supplementary Table 1). Single-insertion mutants were created for 32 sites (amino acid positions: 23, 61, 62, 116, 153, 186, 188, 191, 207, 209, 217, 222, 224, 236, 237, 238, 239, 240, 253, 259, 264, 300, 306, 332, 335, 369, 370, 378, 385, 400, and 401) and then replaced with the domains for which libraries were previously generated. Subsequently, using BsrGI and PstI sites, EGFP was replaced with miRFP670 for all mutants. miRFP670 was amplified from pmiRFP670-N1 using primers 73–74 (Supplementary Table 1), which was a gift from Vladislav Verkhusha (Addgene plasmid # 79987)^69^. The same cloning approach was used to add 3–9 amino acid GSG linkers on either side of Cib81 in the 238 position using primers 85–90 (Supplementary Table 1).

The resting membrane potential assay^54^ was conducted on all aforementioned domain insertion mutants in addition to miRFP670 alone (negative control) and wild-type Kir2.1 (positive control). A total of 400 ng of each mutant was transfected with 6 µl of polyethyleneimine (Polysciences) along with 600 ng of dummy plasmid (pATT-Dest) across two wells of a 24-well dish. For each experiment, wild-type Kir2.1 was transfected as a benchmark and for comparison for mutant function. Cells from each well were detached using 300 µl of accutase (Stemcell Technologies), spun down at 450 ×g three times, and resuspended in 200 µl of Tyrode (125 mM NaCl, 2 mM KCl, 3 mM CaCl_2_, 1 mM MgCl, 10 mM HEPES, and 30 mM glucose, pH 7.3). Bis-[**1**,**3**-dibutylbarbituric acid] trimethine oxonol (DiBAC_**4**_(**3**), Thermo Fisher) was added to a final concentration of 950 nM, and cells were filtered in 5-ml cell strainer tubes (Falcon). DiBAC_**4**_(**3**) was diluted every day to exchange buffers from DMSO to Tyrode. Cells were kept on ice and protected from light in the transfer to the flow cytometry core.

Each sample was run in entirety on a BD Fortessa H0081 flow cytometer. DiBAC_**4**_(**3**) was excited at 488-nm and recorded at 525/50-nm band-pass, and miRFP670 fluorescence was excited at 640-nm and recorded with a 670/30-nm band-pass filter. Cells were gated on side-scattering and forward-scattering areas to separate out whole HEK293FT cells, gated on forward-scattering area and height to separate single cells, then gated on co-expressed miRFP670 to gate out cells that received a plasmid, then a gate was set on the lower 50% of a histogram of wild-type Kir2.1 function, and all mutants percentage of cells in this gate are reported. The analysis was performed in FlowJo 10 (FlowJo, LLC). A representative example of this gating scheme is shown in Supplementary Fig. 13.

### Flow cytometry assay for light modulation of Kir2.1 function

The generation of all single mutants used in the optogenetic switching assay was previously described in the resting membrane potential assay in Methods section. A Cry2-P2A-mKate2 domain was generated using gene fragments (Gen9) amplified with primers 91–96 (Supplementary Table 1) and assembled into the expression vector pEGFPN3 (Invitrogen) using BsmbI-mediated Golden Gate cloning^63^. The Kir2.1(V302M) mutant was generated using Q5 site-directed mutagenesis (NEB).

The light-modulation assay was conducted for Cib81 mutants chosen as representative examples for the various permissibility and functional phenotypes we had observed. In addition, negative controls such as wild-type Kir2.1 and a pore-dead mutant V302M were included. Four micrograms of each mutant, 3 µg of dummy plasmid (pATT-Dest), and 100 µg of Cry2-P2A-mKate2 were transiently transfected using 6 µl of PEI across 16 wells of a 24-well dish at 20% confluency. Cells from each well were detached using 300 μl of accutase, washed three times, and resuspended in 4 ml of Tyrode. DiBAC_**4**_(**3**) was added to a final concentration of 950 nM, and cells were filtered in cell strainer 5-ml tubes (Falcon). Filtered cells were divided into 12 5-ml tubes (300 µl each) and kept on ice and protected from light in the transfer to the flow cytometry core.

Cells expressing each mutant, WT Kir2.1, and Kir2.1(V302M) were challenged by the addition of K-gluconate at different concentrations (5 mM, 10 mM, 15 mM, 25 mM, 40 mM, and 70 mM), with and without illumination (455-nm LED (Thorlabs), 30 s, 100% duty cycle, 100 µW/mm^2^). Each sample was run in entirety on a BD Fortessa H0081 flow cytometer. DiBAC_**4**_(**3**) was excited at 488-nm and recorded with 525/50-nm band-pass and 502-nm long-pass filters, miRFP670 was excited at 640-nm and recorded with a 670/30-nm band-pass filter, and mKate2 was excited at 561-nm and recorded at 610/20-nm band-pass and 595-nm long-pass filters.

Each sample was recorded for 5 min or until completion. Cells were gated on side scattering and forward scattering to separate out whole HEK293FT cells, gated on forward-scattering area and width to separate single cells, and then gated on co-expressed miRFP670 (Kir2.1 mutant) to gate out cells that received a mutant plasmid. For each paired sample (dark and light), a custom gate was created in the non-illuminated sample to include the 15% most hyperpolarized cells (using the flowStats package^70^). The number of events falling into this gate were then compared with the corresponding illuminated sample using the chi-squared test and reported as dissimilarity (Χ^2^, light vs. dark). Dissimilarities at different K^+^ challenges were normalized to correct for photobleaching and averaged. A representative example of this gating scheme is shown in Supplementary Fig. 14.

### Patch-clamp electrophysiology

HEK293FT cells were transiently transfected with Kir2.1 (WT) or Kir2.1 insertion mutant and Cry2-P2A-mKate using PEI. Cells were screened for mKate2 expression using a 565-nm high-power LED (Thorlabs) filtered by a 560/40-nm band-pass filter (Semrock) through a 40X lens. K^+^ currents were recorded 36–48 h post transfection using on-cell patch-clamp electrophysiology. Patches with clear channel activity were stimulated with blue (455-nm) light delivered by a LED (Thorlabs) at 100 µW/mm^2^ for 50 s at 100% duty cycle. Analog signals were filtered (2–5 kHz) using the built-in four-pole Bessel filter of a Sutter Instrument IPA patch-clamp amplifier, digitized, and stored. Bath solution contained 125 mM NaCl, 2 mM KCl, 3 mM CaCl_2_, 1 mM MgCl_2_, 10 mM HEPES, and 30 mM glucose, adjusted to pH 7.3 with NaOH. The pipette solution contained 125 mM K-gluconate, 8 mM NaCl, 0.1 mM CaCl_2_, 0.6 mM MgCl_2_, 1 mM EGTA, 10 mM HEPES, 4 mM Mg-ATP, and 0.4 mM Na-GTP, adjusted to pH 7.3 with KOH. Osmolarity was adjusted to 295–300 mOsm with sucrose. Electrodes were drawn from borosilicate patch glass (Warner Instruments) to a resistance of 2–6 MΩ. Data analysis was done using custom R scripts.

### Reporting Summary

Further information on experimental design is available in the Nature Research Reporting Summary linked to this article.

## Supplementary information

Supplementary Information

Supplementary Info File—reporting summary

## Data Availability

Data supporting the findings of this paper are available from the corresponding author upon reasonable request. The source data underlying Figs. 2–4, 6a, 7b, and Supplementary Figs. 3–10 are provided as a Source Data file in the Sequence Raw Archive (SRA—https://www.ncbi.nlm.nih.gov/sra) and accession code for the data is [PRJNA506141].
