# Supplementary material for: Domain insertion permissibility-guided engineering of allostery in ion channels
Source: Nat Commun. 2019 Jan 17;10:290. doi: 10.1038/s41467-018-08171-0 (PMC6336875; doi:10.1038/s41467-018-08171-0)
Supplement: Supplementary file 1 — Supplementary Information [file 41467_2018_8171_MOESM1_ESM.pdf]

Supplementary Materials for

Domain Insertion Permissibility-Guided Engineering of Allostery in Ion Channels

Willow Coyote-Maestas[1], Yungui He[2], Chad L. Myers[3] & Daniel Schmidt[2*]

[1]Dept. of Biochemistry, Molecular Biology & Biophysics

[2]Dept. of Genetics, Cell Biology & Development

[3]Dept. of Computer Science and Engineering, University of Minnesota,

321 Church Street SE, 6-160 Jackson, Minneapolis, MN, United States of America

Correspondence and requests for materials should be addressed to D.S. (email:schmida@umn.edu)

This PDF file includes:

Supplementary Figures 1 to 14
Supplementary Table 1

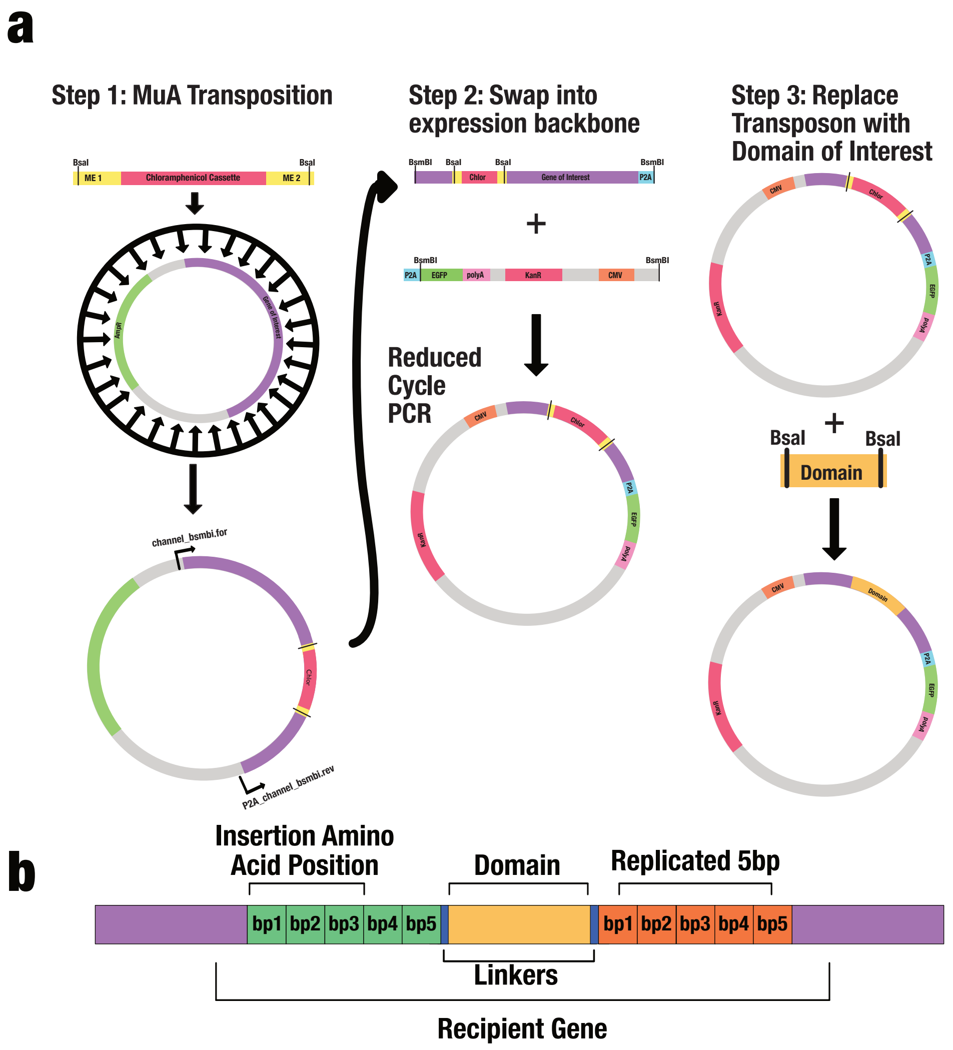

Supplementary Figure 1: Insertion Library Construction. (a) Libraries were generated in three cloning and selection steps. (1) Select for a MuA transposase-delivered engineered MuA transposon with a chloramphenicol antibiotic cassette in a plasmid carrying Kir2.1. Flanking the antibiotic cassette are the beginnings and ends of flexible linkers with Golden Gate compatible BsaI type IIS restriction sites. (2) Reduced-cycle PCR amplify, add on Golden Gate compatible BsmBI type IIS restriction sites, and size separate channel genes with inserted transposons from those without transposons. Insert channel gene into a mammalian expression vector in-frame with a P2A-EGFP cassette. (3) Replace the transposon with a PCR amplified domain of interest with complementary BsaI sites and linkers using BsaI-mediated Golden Gate cloning. **(b)** Architecture of a domain insertion position: At the position of the domain insertion the five positions upstream are replicated on the other side of the transpositions; domain insertion positions are identified as the last full codon coding for an amino acid or in other words that corresponding to the amino acid coded by bp1-3 of the replicated sequence. Domains are inserted with linkers to bring it into frame after insertion at +2 reading frame relative to the coding sequence.

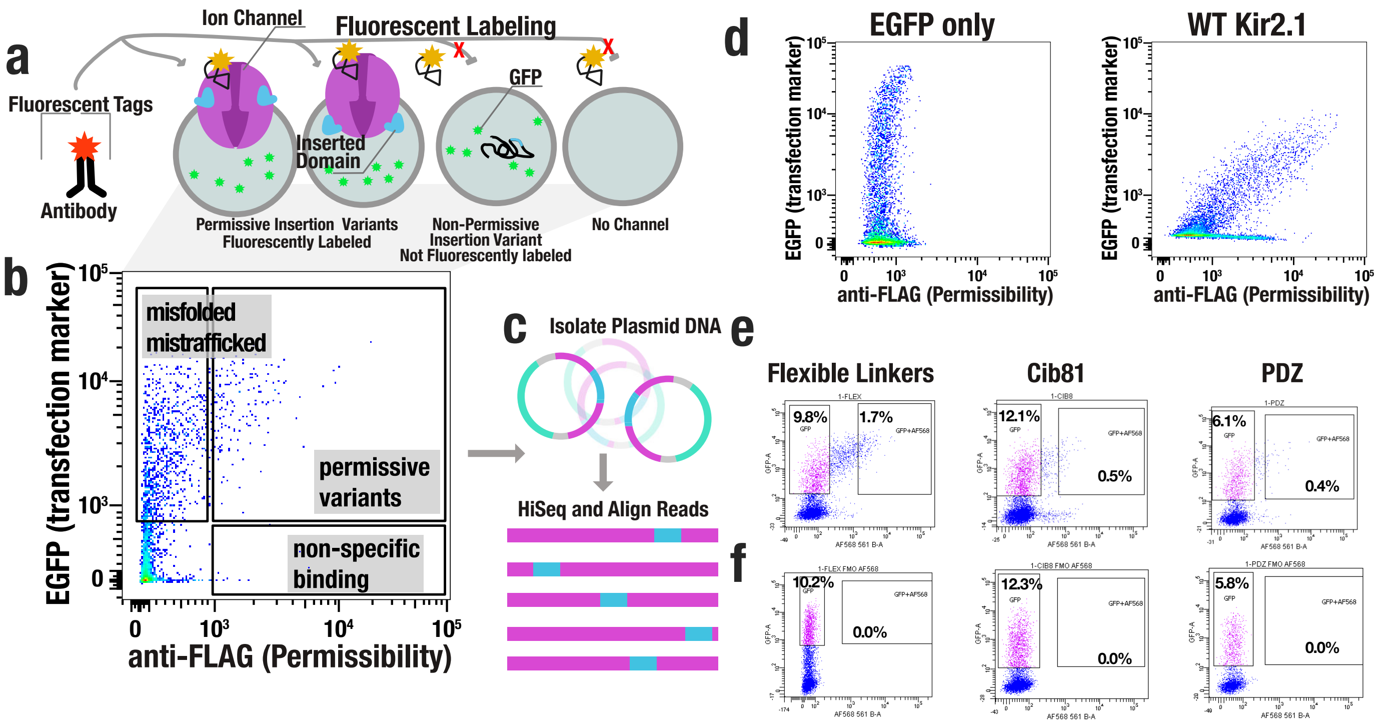

Supplementary Figure 2: Permissibility Assay. (a) Domain insertion libraries are transiently expressed in HEK293FT cells and labeled with anti-FLAG Alexa568. (**b**) Cells are isolated by flow sorting into two populations: surface expressed (permissive insertion variants) and non-surface expressed (non-permissive) insertion variants. (**c**) Plasmid DNA is isolated from each population and subject to HiSeq. (**d**) Labeling controls (only GFP and WT Kir2.1-P2A-EGFP) expressed in HEK293FT to demonstrate antibody labeling. (**e**) Examples for each anti-FLAG labeled sorted samples ($GSAG_{x2}$ and $GSAG_{x3}$, Cib81 and PDZ) expressed in HEK293FT cells with gates that were used for sorting. (**f**) Identical samples as in (**e**) without anti-FLAG labeling to guide setting gates.

Cib81

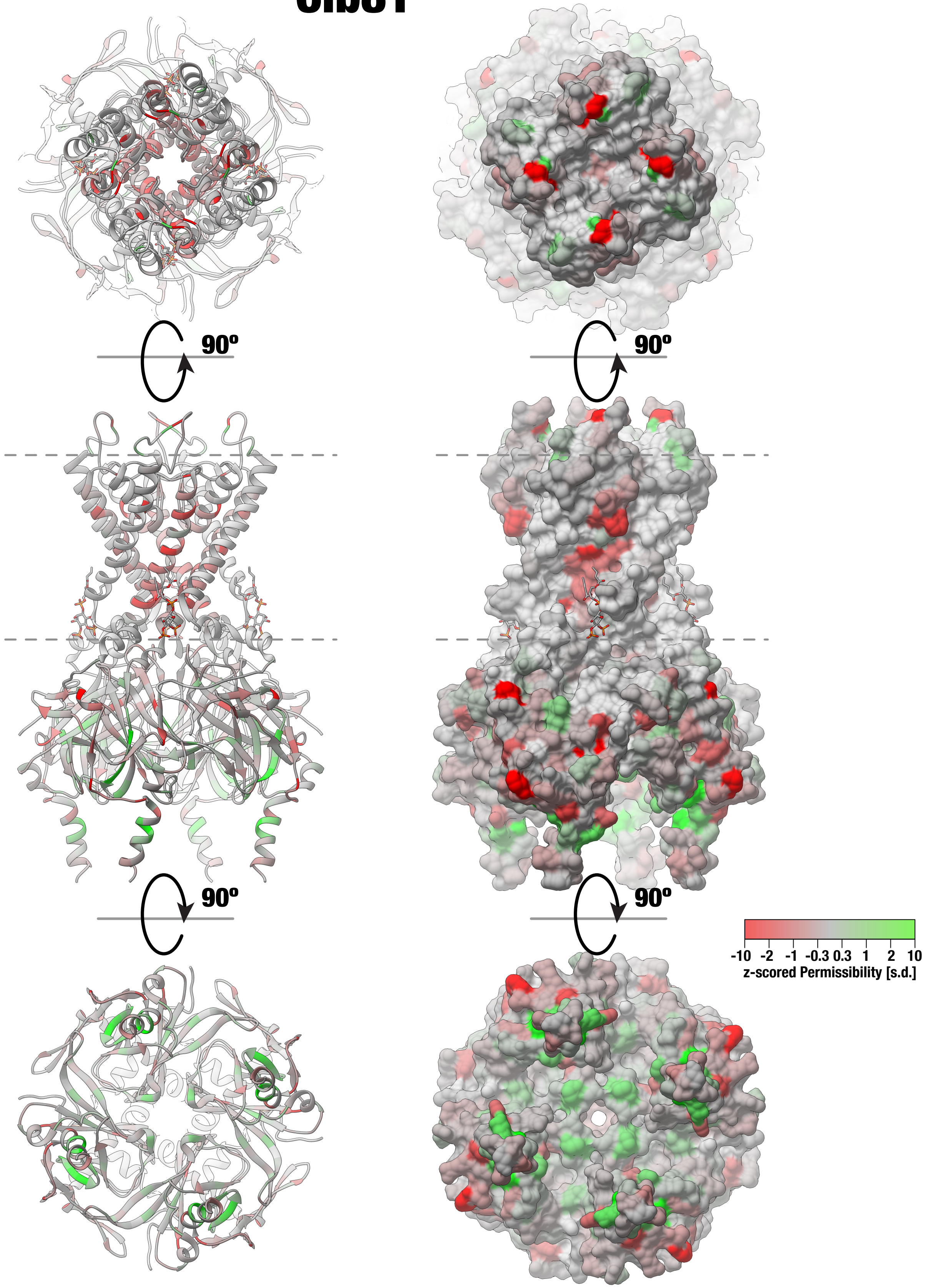

Supplementary Figure 3: Domain Insertion Permissibility. Permissibility data for inserting Cib81 is mapped on the crystal structure of chicken Kir2.2 (PDB 3SPI) displayed as a ribbon (left) or surface model (right). Dashed lines indicate plasma membrane boundaries.

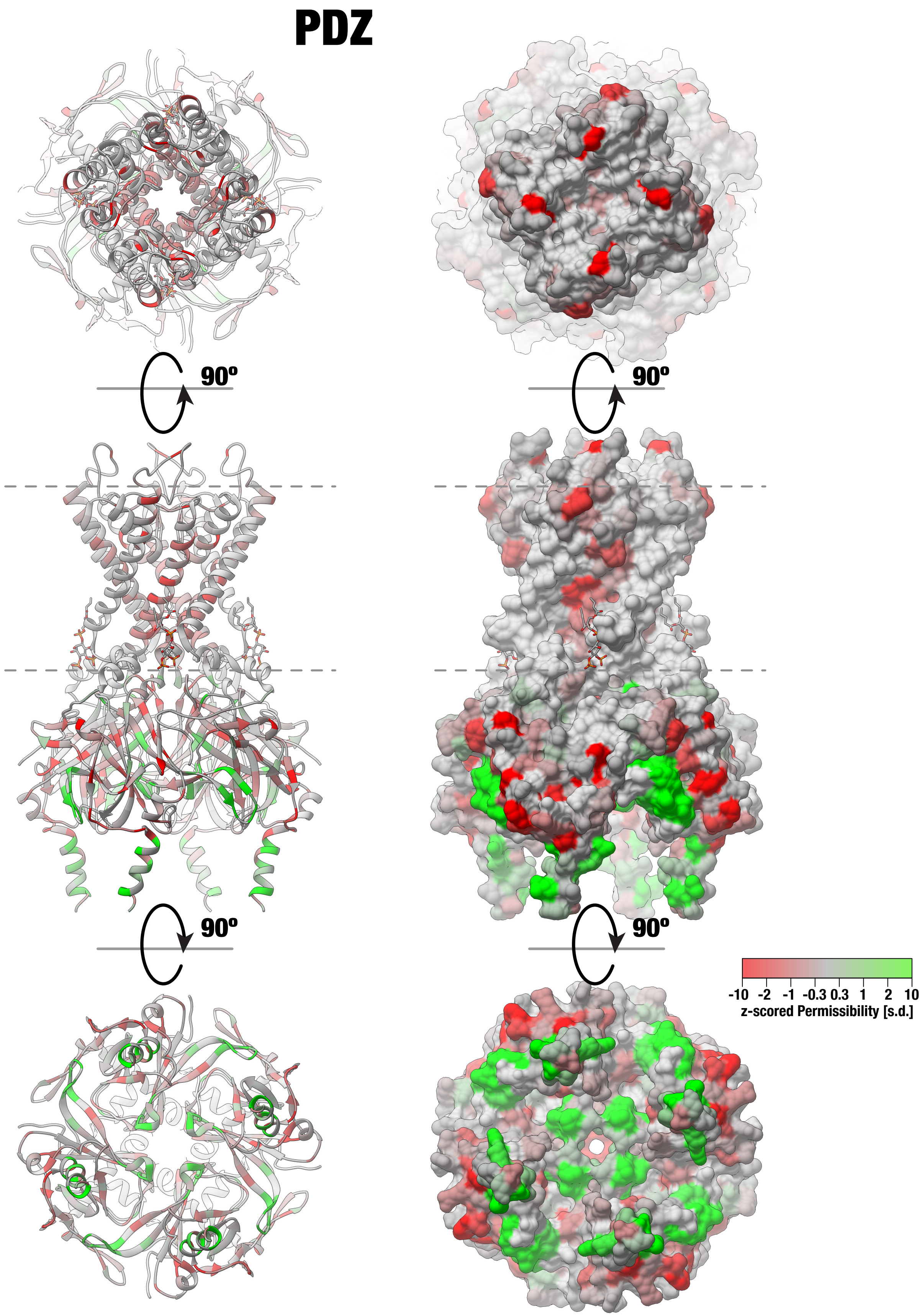

Supplementary Figure 4: Domain Insertion Permissibility. Permissibility data for inserting PDZ is mapped on the crystal structure of chicken Kir2.2 (PDB 3SPI) displayed as a ribbon (left) or surface model (right). Dashed lines indicate plasma membrane boundaries.

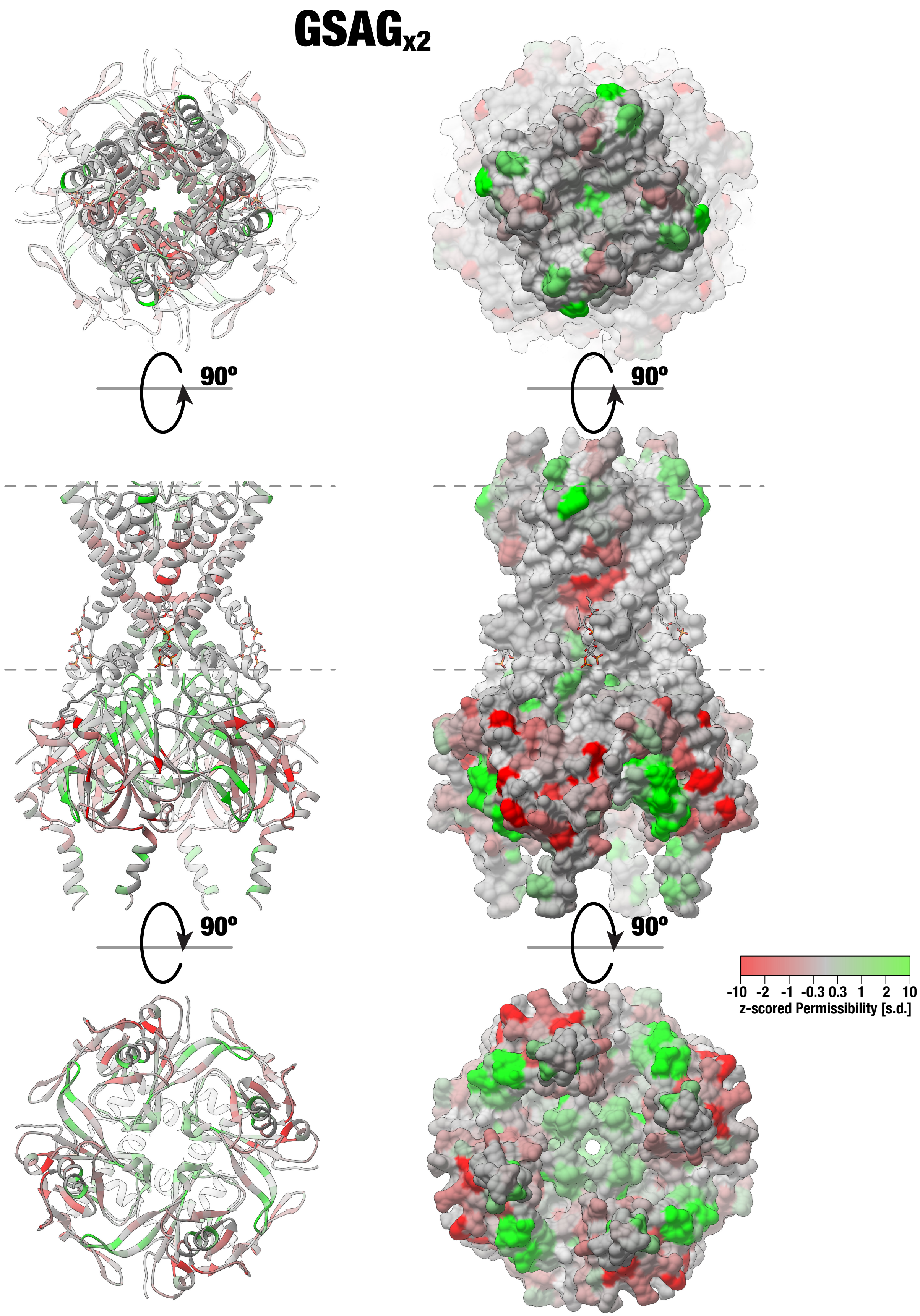

Supplementary Figure 5: Domain Insertion Permissibility. Permissibility data for inserting $GSAG_{x2}$ linkers is mapped on the crystal structure of chicken Kir2.2 (PDB 3SPI) displayed as a ribbon (left) or surface model (right). Dashed lines indicate plasma membrane boundaries.

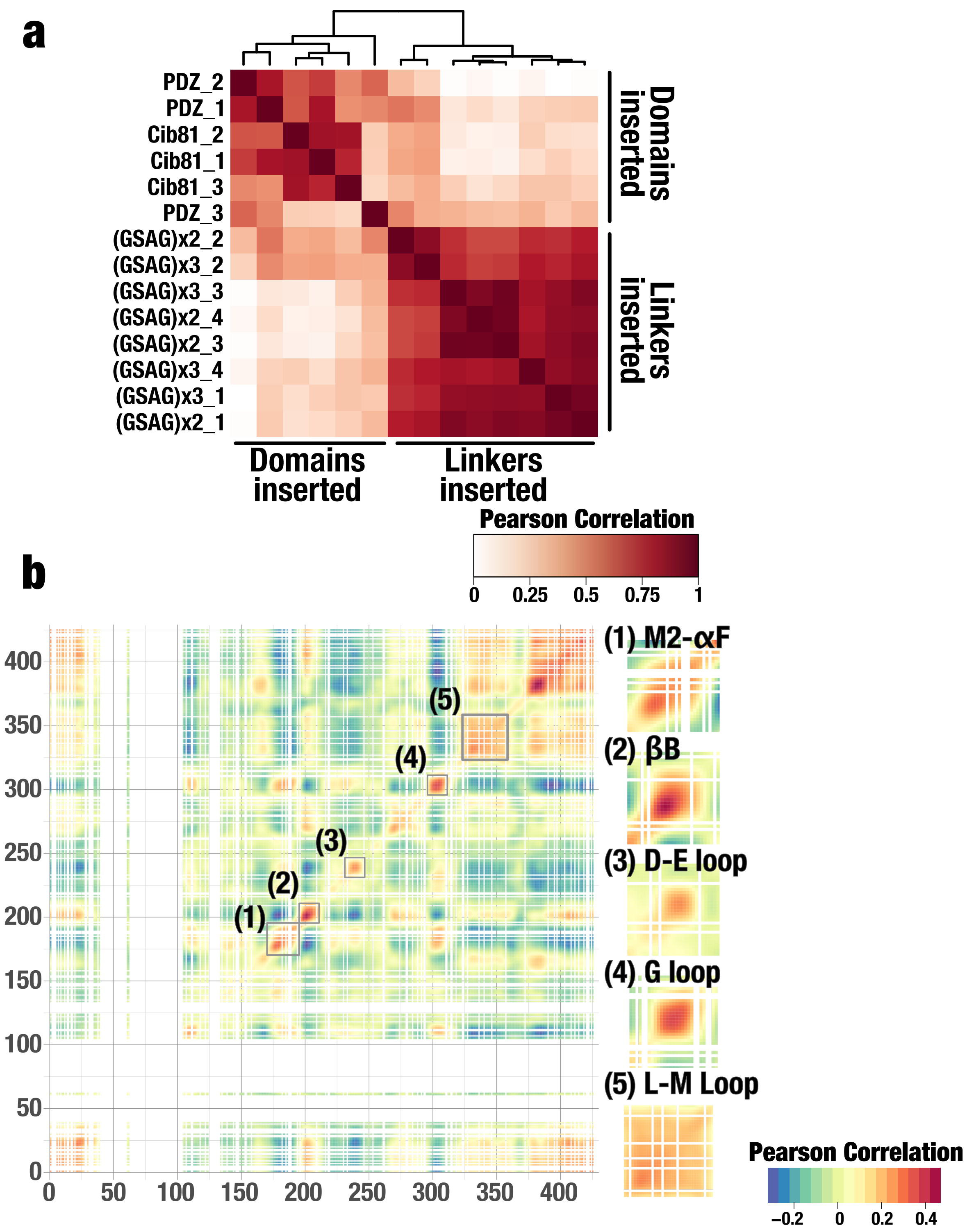

Supplementary Figure 6: Pearson correlation of biological replicates and site-specific permissibility correlations. (a) Hierarchical clustering of all individual permissibility datasets based on domain structure and length. Biological replicates show a high degree of reproducibility. As expected, datasets for structured domains (Cib81 and PDZ) cluster discretely with themselves than with each other whereas flexible linkers cluster indiscriminately between each other. **(b)** In Kir2.1, PDZ permissibility is highly correlated in (1) M2-αF – the PIP_2 binding site, (2) the βB loop where ATP binds in Kir6.2, (3 & 5) the βD-βE and βL-βM loops where Gβγ binds in GIRK, as well as (4) the G loop involved in channel gating. White indicated sites with incomplete data.

a

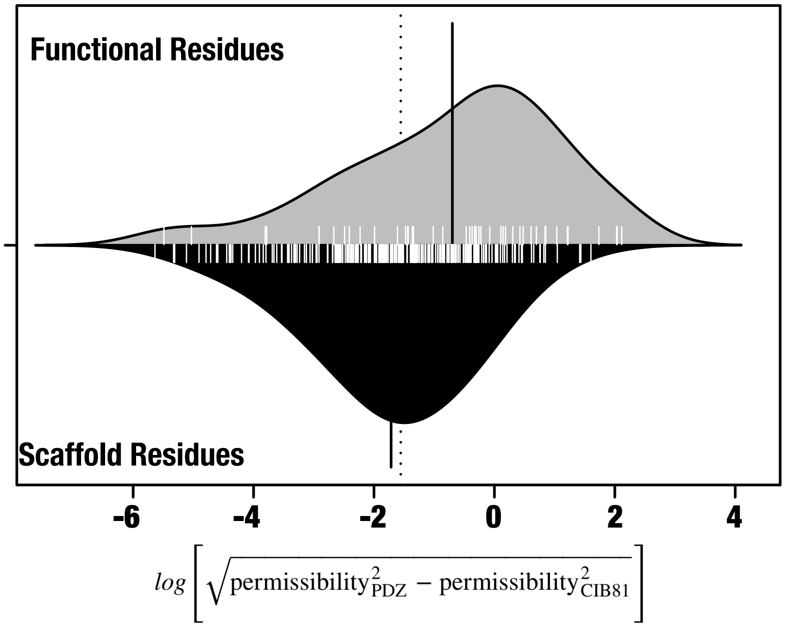

Supplementary Figure 7: Functionally important sites are more likely to be differentially permissive. (a) Kir2.1 residues are termed functional if they are involved in Kir2.1 gating (e.g. PIP_2 binding site) or a residues that corresponds to those involved in gating of a homolog (e.g. Gβγ-binding in GIRK). Beanplots for the calculated per-residue log-transformed domain insertion permissibility difference in PDZ and CIB81 datasets. Data for residues involved in function in Kir2.1 or related homologs is shown in grey; data for scaffold residues not involved in function is shown in black. White beanlines indicate calculated differences for individual residues. Solid vertical lines indicates then bean average for each group, while the vertical dashed line indicates the total average of for both groups.

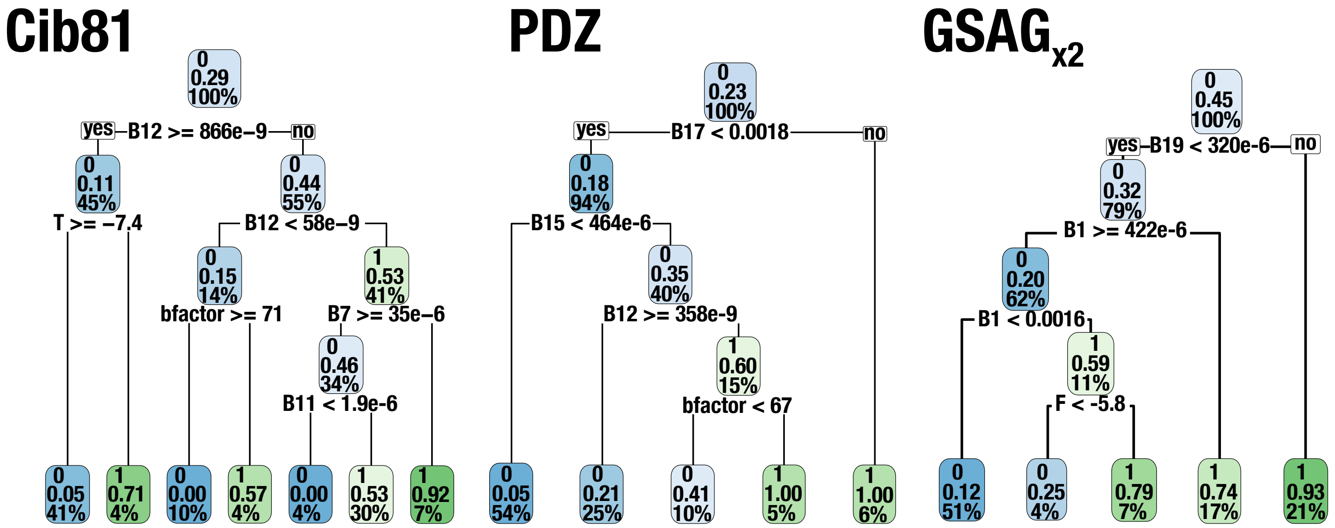

Supplementary Figure 8: Decision Trees. Decision trees were trained on calculated conservation, static structural and dynamic protein properties to predict binarized Cib81, PDZ and $GSAG_{x2}$ permissibility. Features and cutoffs for each node are selected based on a 'complexity parameter' which penalizes uninformative complexity. All trees were restricted to a maximum depth of four and evaluated using 10-fold cross-validation. Decision tree leaves can be read as: the top-most number refers to the leaf class (0–not permissive and 1–permissive), next are percentage of permissive samples within the leaf that fall within the class, and percentage of all data that are within the leaf. Color intensity refers to purity of sample, at each leaf (fraction of non-permissive-to-permissive withh a blue-to-green color scale). As can be seen in the models, most of the nodes use dynamic properties. This suggests that dynamic properties are more discriminative compared to other property classes.

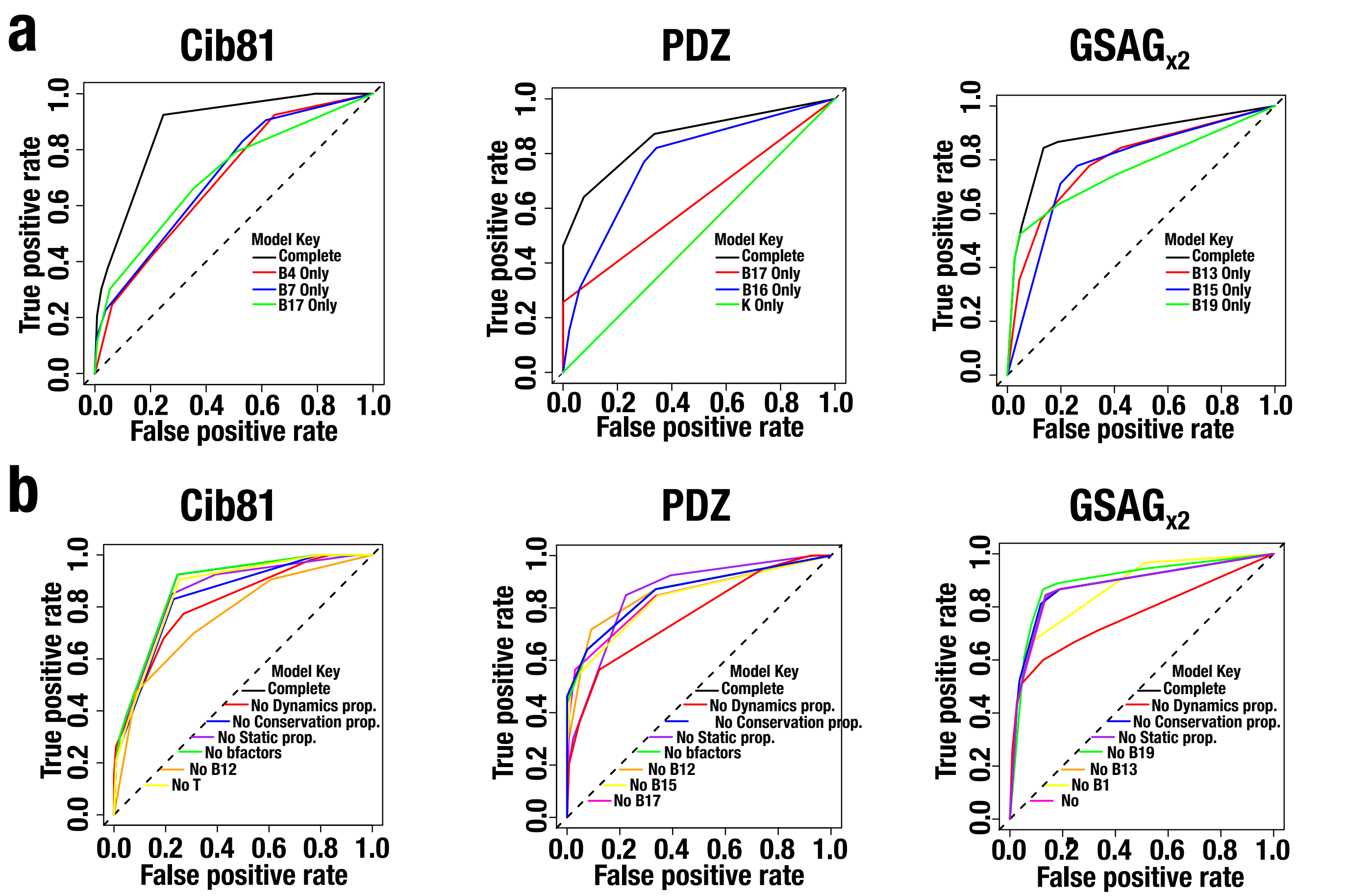

Supplementary Figure 9: Decision trees trained on limited numbers of properties. (a) Decision trees were trained using the top three Spearman co-efficient correlated properties individually using the same model parameters as before (a maximum depth of four and cross-validated ten times). Model performance was compared using receiver operator characteristic (ROC) curves. All models trained on single properties perform worse than models trained all properties. This suggests that multiple interacting properties determine permissibility. Interestingly, the predicted effect of any amino acid mutated to lysine was highly linearly correlated with PDZ permissibility. Nevertheless, using it alone was not sufficient to predict PDZ permissibility. On the other hand, normal modes –a dynamic feature– were able to predict PDZ permissibility when used individually. **(b)** Decision trees were trained (maximum depth of four and 10-fold cross-validated) with individual properties or entire property classes withheld. Model performance was compared using ROC curves. In every case, removing all computed conservation and static structural properties had little effect on model performance. In contrast, removing dynamics based properties was detrimental to permissibility predictions. Apart from two examples (Cib81~B12 and $GSAG_{x2}$~B1) removing individual properties were not substantially impactful on model performance suggesting that there is redundancy in computed properties. Overall, all decision trees reinforce the idea that (1) dynamics are the most informative and closest correlated protein properties to permissibility and (2) interactions among protein properties are important in predicting permissibility.

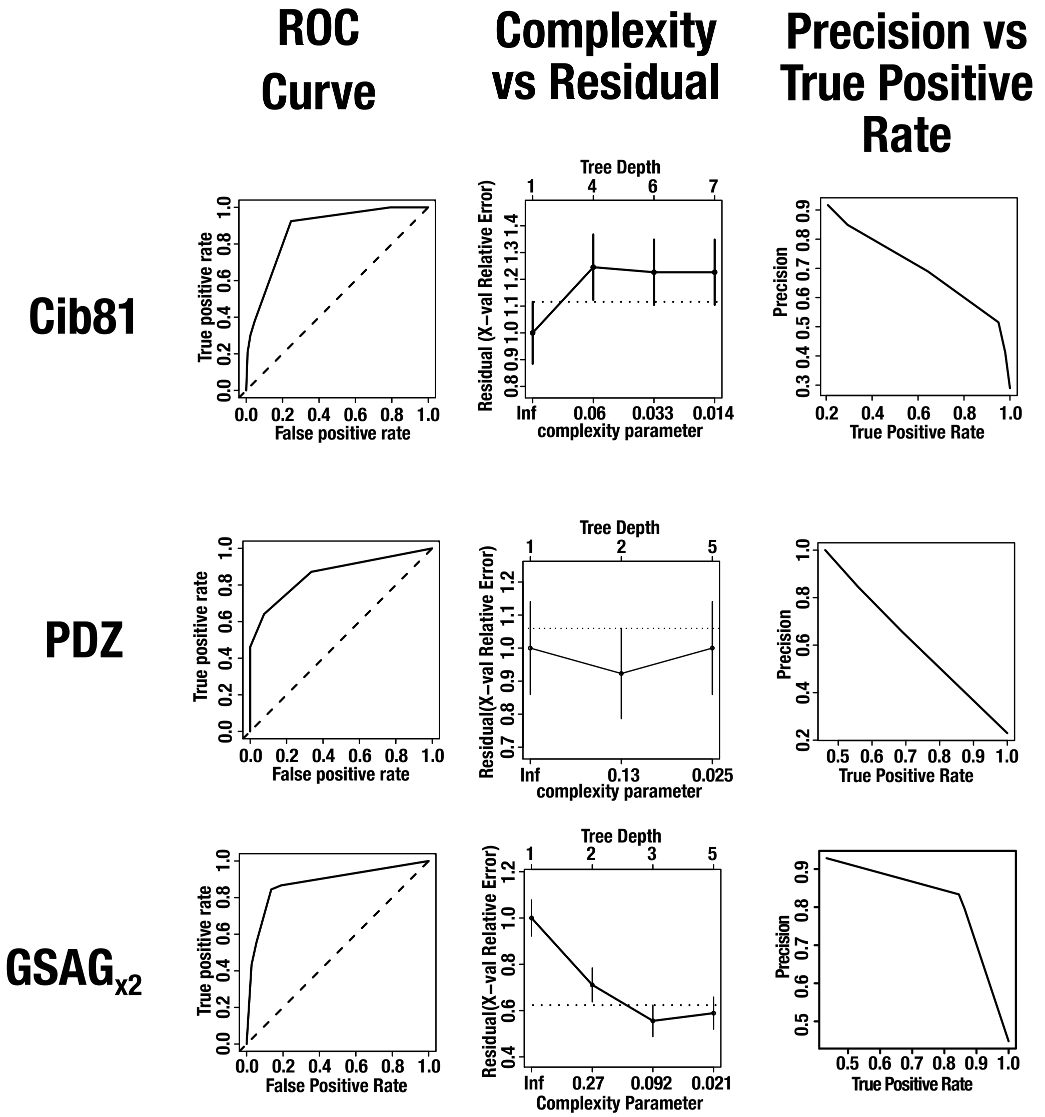

Supplementary Figure 10: Decision Tree Model Performance. (Left Panels) Cib81, PDZ, and GSAG$_{x2}$ decision tree performance using different criteria. All model criteria are derived by testing the model on test data withheld from training data. Receiver operator characteristic (ROC) curves also shown in **Figure 4b** are the proportion of positive data that are predicted positive (true positive rate) plotted against the proportion of predicted negative data that are predicted positive (false positive rate) at varying model cut-offs. The dotted line represents the performance of a random model. ROC curves demonstrate that as threshold are changed, true positives increase at the expense of increasing false positives. All models perform far better than random. (Center Panels) Complexity vs. residual plots show the difference between predicted and actual data (residual) plotted against the tree depth with the amount that splitting a node improved model performance (complexity parameter) used for trimming trees also noted. The dotted line represents the ideal threshold residual for model performance and error bars are s.e.m. (n=10). These plots demonstrate that increasing tree depth, and therefore tree complexity, has diminishing impact on improving model performance. (Right panel) Precision vs. true positive rate plots show proportion of positive predicted data that are positive (precision) plotted against the true positive rate at varying model cut-offs. Precision-true positive rate curves show that as more true positives are predicted, more negative data are predicted as false positive. A random model would be a flat horizontal line. All models based on all parameters perform far better than random.

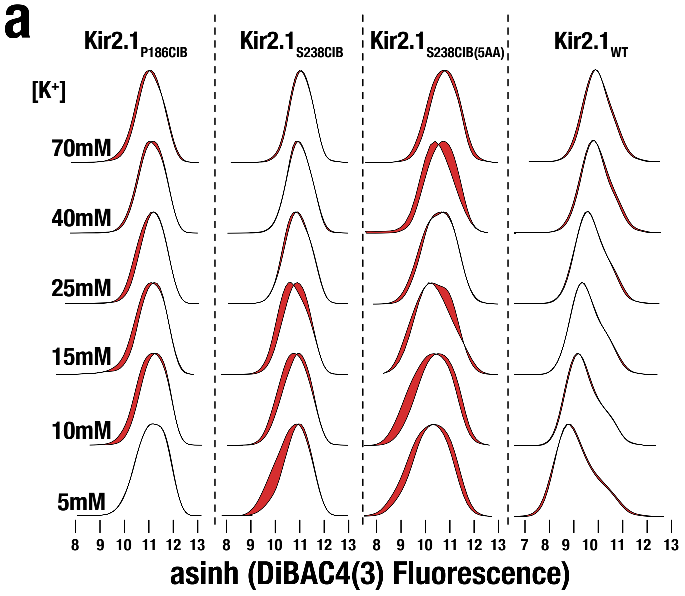

Supplementary Figure 11: Light-modulated Kir2.1 Variants. (a) Representative examples showing the difference (indicated by red areas) in population-level DiBAC**4(3)** fluorescence after depolarization with increasing amounts of external K^+ (indicated left) with and without blue light illumination for HEK293FT cells expressing the indicated Cib81 insertion mutant or WT channel and Cry2.

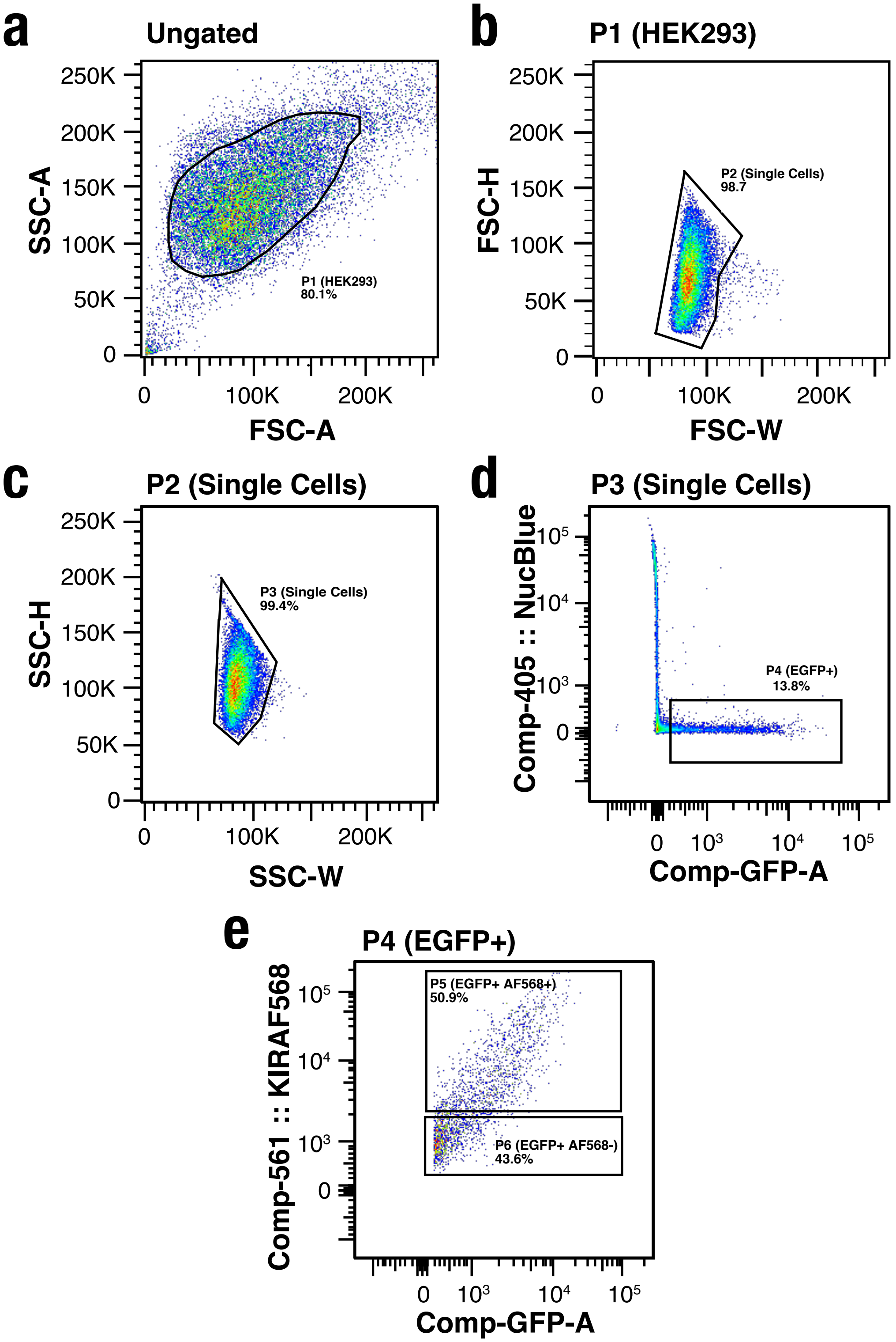

Supplementary Figure 12: Permissibility Gating Scheme. (a) Cells are gated on side and forward scattering area to select whole HEK293 cells. **(b-c)** Forward and side scattering height and width are gated to select single cells. (**d**) Transfected cells are gated based on EGFP signal (Comp-GFP-A). **(e)** Finally, EGFP high / Label low and EGFP high / Label high populations are gated based on EGFP (Comp-GFP-A) and Alexa fluorophore 568 fluorescence (secondary antibody, Comp-561).

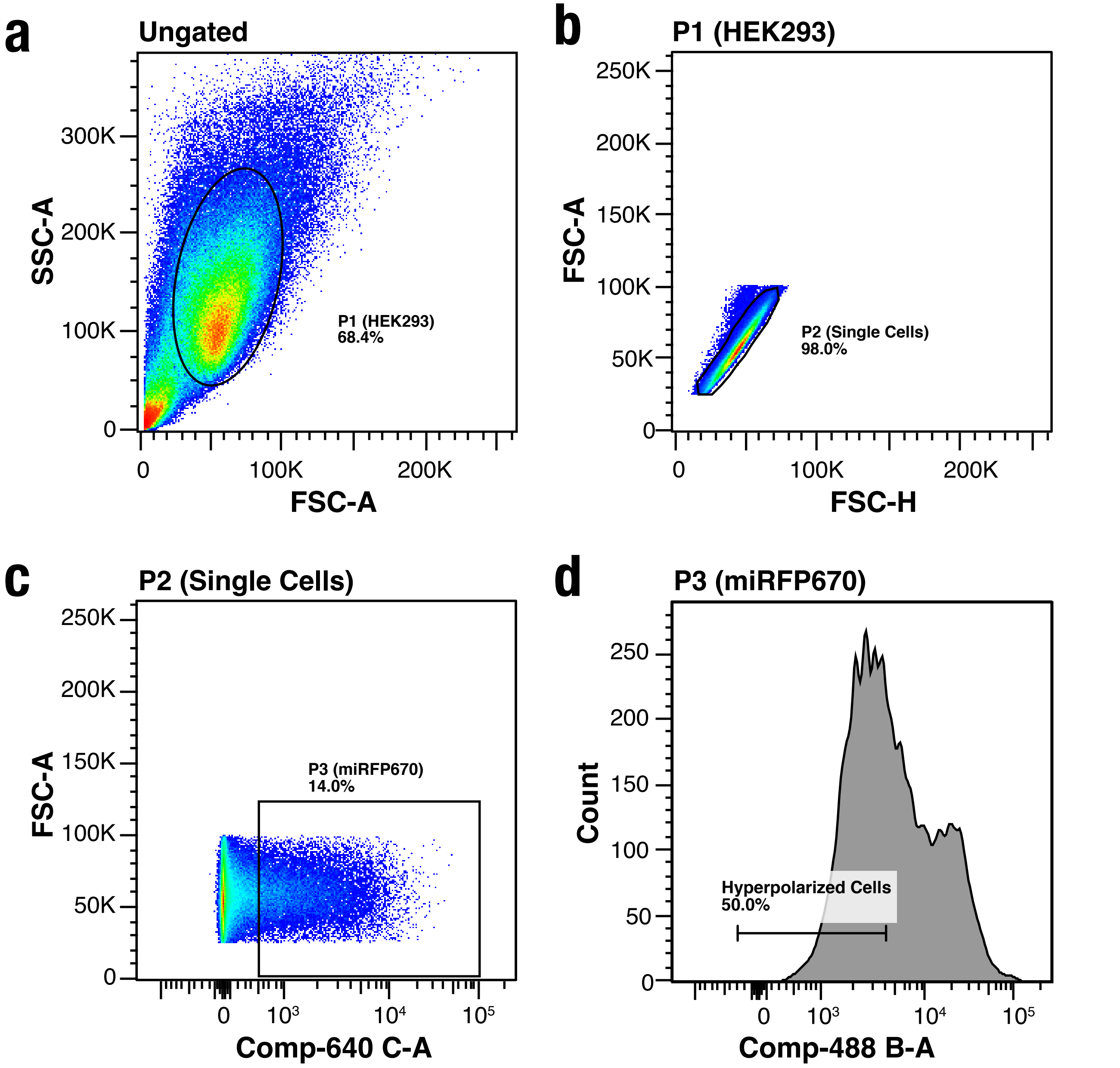

Supplementary Figure 13: Voltage Assay Gating Scheme. (a) Cells are gated on side and forward scattering area to select whole HEK293 cells. (b) Next, cells are gated on forward scattering area and height to select single cells. **(c)** Transfected cells are gated based on miRFP670 signal (Comp-640 B-A). **(d)** Finally, a gate was set on the lower 50% of cells (corresponding to more hyperpolarized cells) based on DiBAC4(3) fluorescence (Comp-488 B-A) in wildtype Kir2.1 samples. The same gate was applied to all mutants and percentage of cells in this gate are reported.

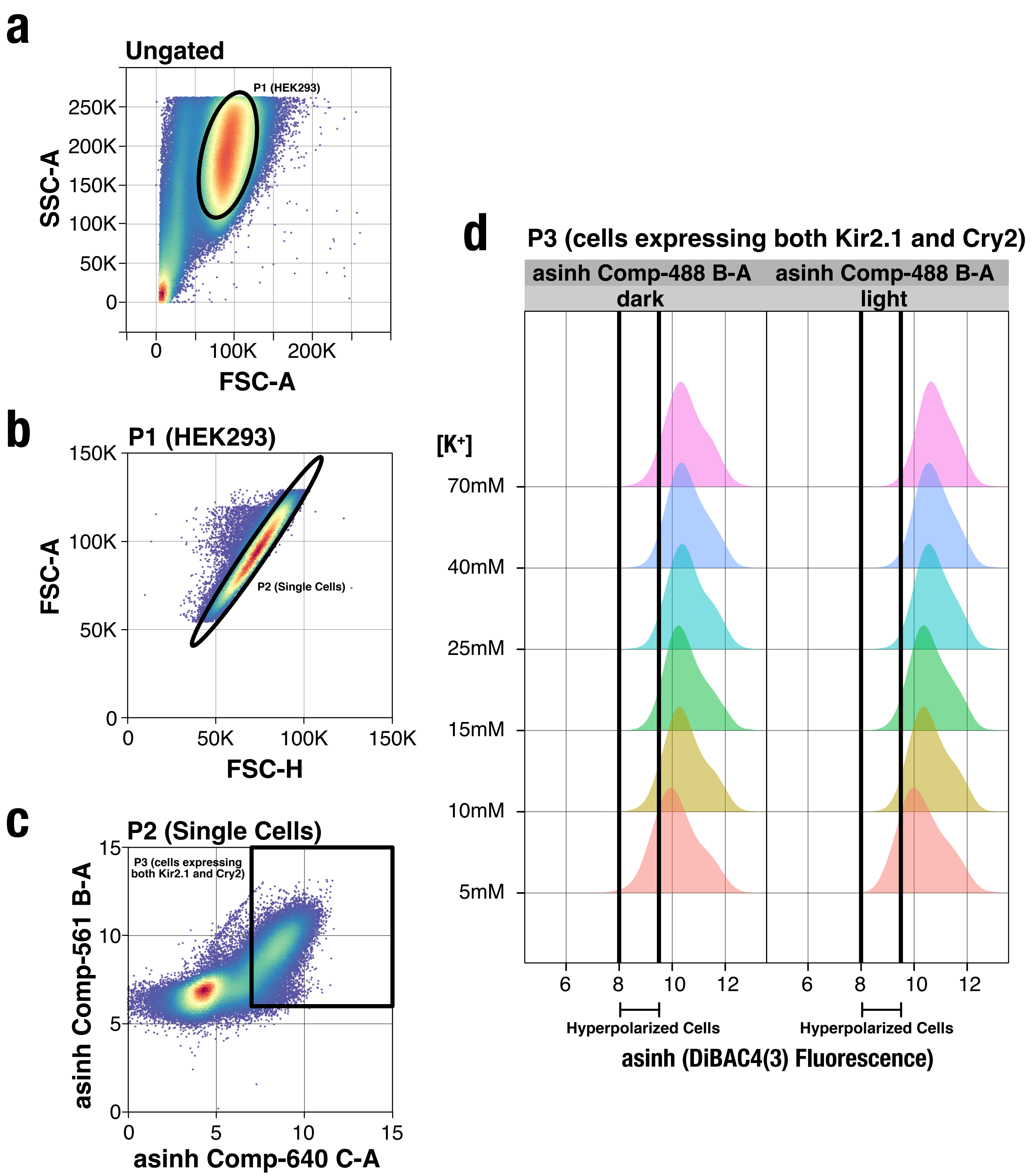

Supplementary Figure 14: Light-Switching Gating Scheme. (a) Cells are gated on side and forward scattering area to select whole HEK293 cells. **(b)** Next, cells are gated on forward scattering area and height to select single cells. **(c)** Double-transfected cells (expressing both Cry2 and Kir2.1) are gated based on mKate2 (Comp-561 B-A) and miRFP670 signal (Comp-640 C-A), respectively. **(d)** Finally, for a given mutant (e.g., wildtype Kir2.1) and a given K^+ challenge, a custom gate was created in the non-illuminated (dark) sample corresponding to the 15% most hyperpolarized cells (reported by DiBAC**4(3)**, Comp-488 B-A). The number of events falling into this gate were compared to the corresponding illuminated sample using the Chi-Squared test and reported as Dissimilarity (χ^2, light vs. dark).

Supplementary Table 1

primer index	Primer Name	Sequence
1	kir2.1_patt.for	GCAGCTaagcttGCATgaagacatccgGCCACCATGGGA
2	kir2.1_patt.rev	GCGGATcctaggTGATgaagacATTGGTCCTGGATTTTCCTCCA
3	kir2.1_cmv.for	gacatacCGTCTCctccgGCCACCATGGGat
4	kir2.1_cmv.rev	gcagagCGTCTCTTGGTCCTGGATTTTCCTCCACATCACC
5	cmv_backbone.for	GACAGCAGCGTCTCgACCAatggtgagcaagggcgag
6	cmv_backbone.rev	acgtcagCGTCTCAcggatctgacggttcactaaacc
7	gfp_cmv.for	taagcagagctggtttagtgaaccgtcagatccgaGAAGACtaCCGGtaagacagcagac
8	gfp_cmv.rev	atttgtaaccattataagctgcaataaacaagttttacttgtacagctcgtccatgcc
9	gfp_cmv_back.for	acgagctgtacaagtaaaacttgtttattgcagcttataatggttacaaa
10	gfp_cmv_back.rev	tcttaCCGGtaGTCTTCtcggatctgacggttcactaaaccag
11	kir2.1_flag_q5.for	GATGATGATAAATCCAAGGTATCCAAGGCCTGTG
12	kir2.1_flag_q5.rev	GCATGGAGATTTGGACACCGATTATAAAGA
13	kir2.1_23_q5_for	cttacgcatttcggtctccgcgtcaaccatGGCTGTAGCCAACGGTTT
14	kir2.1_23_q5_rev	gagaaaataccgcatctcggtctccgatgcaATGGTTGCCAGTTTCATCC
15	kir2.1_61_q5_for	cttacgcatttcggtctccgcgtcagtgggGGAAAAGGGTCAACGGTAC
16	kir2.1_61_q5_rev	gagaaaataccgcatctcggtctccgatgcaCCCACGTTTATGAACTGC
17	kir2.1_62_q5_for	cttacgcatttcggtctccgcgtcaggggaAAAGGGTCAACGGTACTTG
18	kir2.1_62_q5_rev	gagaaaataccgcatctcggtctccgatgcaTCCCCCACGTTTATGAAC
19	kir2.1_116_q5_for	cttacgcatttcggtctccgcgtcagattaTAAAGATGATGATGATAAATCC
20	kir2.1_116_q5_rev	gagaaaataccgcatctcggtctccgatgcaTAATCGGTGTCCAAATCTC
21	kir2.1_153_q5_for	cttacgcatttcggtctccgcgtcagaatgCCCAATAGCCGTTTTCATG
22	kir2.1_153_q5_rev	gagaaaataccgcatctcggtctccgatgcaCATTCGTCTGTGACGCATC
23	kir2.1_186_q5_for	cttacgcatttcggtctccgcgtcacccaaAAAAAGAAATGAGACATTGGTTTTC
24	kir2.1_186_q5_rev	gagaaaataccgcatctcggtctccgatgcaTTGGGCTTAGCCATTTTTG
25	kir2.1_188_q5_for	cttacgcatttcggtctccgcgtcaaaaagAAATGAGACATTGGTTTTCAG
26	kir2.1_188_q5_rev	gagaaaataccgcatctcggtctccgatgcaCTTTTTTTGGGCTTAGCC
27	kir2.1_191_q5_for	cttacgcatttcggtctccgcgtcagagacATTGGTTTTCAGTCACAAC
28	kir2.1_191_q5_rev	gagaaaataccgcatctcggtctccgatgcaGTCTCATTTCTTTTTTTGGG
29	kir2.1_207_q5_for	cttacgcatttcggtctccgcgtcaaagctGTGCCTCATGTGGAGGGT
30	kir2.1_207_q5_rev	gagaaaataccgcatctcggtctccgatgcaAGCTTGCCATCCCTCATG
31	kir2.1_209_q5_for	cttacgcatttcggtctccgcgtcatgcctCATGTGGAGGGTGGGCAAT
32	kir2.1_209_q5_rev	gagaaaataccgcatctcggtctccgatgcaAGGCACAGCTTGCCATCC
33	kir2.1_217_q5_for	cttacgcatttcggtctccgcgtcactgagAAAGTCCCACCTCGTAGAG
34	kir2.1_217_q5_rev	gagaaaataccgcatctcggtctccgatgcaCTCAGATTGCCCACCCTC
35	kir2.1_222_q5_for	cttacgcatttcggtctccgcgtcactcgtAGAGGCCCATGTACGAGC
36	kir2.1_222_q5_rev	gagaaaataccgcatctcggtctccgatgcaACGAGGTGGGACTTTCTC
37	kir2.1_224_q5_for	cttacgcatttcggtctccgcgtcagaggcCCATGTACGAGCACAACTG
38	kir2.1_224_q5_rev	gagaaaataccgcatctcggtctccgatgcaGCCTCTACGAGGTGGGAC
39	kir2.1_236_q5_for	cttacgcatttcggtctccgcgtcaataacTTCAGAAGGAGAGTACATAC
40	kir2.1_236_q5_rev	gagaaaataccgcatctcggtctccgatgcaGTTATGCGTGATTTCAGC
41	kir2.1_237_q5_for	cttacgcatttcggtctccgcgtcaacttcTACATACCACTCGATCAG
42	kir2.1_237_q5_rev	gagaaaataccgcatctcggtctccgatgcaGAAGTTATGCGTGATTTC
43	kir2.1_238_q5_for	cttacgcatttcggtctccgcgtcatcagaAGGAGAGTACATACCACTC
44	kir2.1_238_q5_rev	gagaaaataccgcatctcggtctccgatgcaTCTGAAGTTATGCGTGATTTC
45	kir2.1_240_q5_for	cttacgcatttcggtctccgcgtcaggagaGTACATACCACTCGATCAG
46	kir2.1_240_q5_rev	gagaaaataccgcatctcggtctccgatgcaTCTCCTTCTGAAGTTATGC
47	kir2.1_253_q5_for	cttacgcatttcggtctccgcgtcattcgaTAGCGGCATTGACAGGAT
48	kir2.1_253_q5_rev	gagaaaataccgcatctcggtctccgatgcaTCGAAGCCCACATTGATATC

49	kir2.1_259_q5_for	cttacgcatttcggtctccgcgtcagacagGATCTTTCTCGTTAGCCC
50	kir2.1_259_q5_rev	gagaaaataccgcatctcggtctccgatgcaCTGTCAATGCCGCTATCG
51	kir2.1_264_q5_for	cttacgcatttcggtctccgcgtcagttagCCCAATCACCATCGTCCA
52	kir2.1_264_q5_rev	gagaaaataccgcatctcggtctccgatgcaCTAACGAGAAAGATCCTGTC
53	kir2.1_300_q5_for	cttacgcatttcggtctccgcgtcagggatGGTAGAGGCCACCGCTAT
54	kir2.1_300_q5_rev	gagaaaataccgcatctcggtctccgatgcaATCCCTTCCAGTATAACGACAATTTC
55	kir2.1_306_q5_for	cttacgcatttcggtctccgcgtcagctatGACAACCCAATGTCGAAGTAG
56	kir2.1_306_q5_rev	gagaaaataccgcatctcggtctccgatgcaATAGCGGTGGCCTCTACC
57	kir2.1_332_q5_for	cttacgcatttcggtctccgcgtcagaggaGAAGCACTATTATAAGGTCG
58	kir2.1_332_q5_rev	gagaaaataccgcatctcggtctccgatgcaTCCTCAAACAAGACAGGTTC
59	kir2.1_335_q5_for	cttacgcatttcggtctccgcgtcacactaTTATAAGGTCGACTACTC
60	kir2.1_335_q5_rev	gagaaaataccgcatctcggtctccgatgcaTAGTGCTTCTCCTCAAAC
61	kir2.1_369_q5_for	cttacgcatttcggtctccgcgtcatctaaCGCAAACTCTTTCTGTTAC
62	kir2.1_369_q5_rev	gagaaaataccgcatctcggtctccgatgcaTTAGACAGAATGTACTTCTTC
63	kir2.1_370_q5_for	cttacgcatttcggtctccgcgtcaaacgcAAACTCTTTCTGTTACGAG
64	kir2.1_370_q5_rev	gagaaaataccgcatctcggtctccgatgcaGCGTTAGACAGAATGTAC
65	kir2.1_378_q5_for	cttacgcatttcggtctccgcgtcaaacgaAGTAGCTCTCACATCAAAG
66	kir2.1_378_q5_rev	gagaaaataccgcatctcggtctccgatgcaTCGTTCTCGTAACAGAAAG
67	kir2.1_385_q5_for	cttacgcatttcggtctccgcgtcaaaggaAGAAGAAGAAGATTCAGAGAAC
68	kir2.1_385_q5_rev	gagaaaataccgcatctcggtctccgatgcaTCCTTTGATGTGAGAGCTAC
69	kir2.1_400_q5_for	cttacgcatttcggtctccgcgtcaagcacCGACTCTCCTCCCGGCAT
70	kir2.1_400_q5_rev	gagaaaataccgcatctcggtctccgatgcaGTGCTGGTTGACTCGGGAAC
71	kir2.1_401_q5_for	cttacgcatttcggtctccgcgtcaaccgaCTCTCCTCCCGGCATTGA
72	kir2.1_401_q5_rev	gagaaaataccgcatctcggtctccgatgcaTCGGTGCTGGTTGACTCG
73	miRFP670_for	CTTCATCTGCAGTCAATGGGTCAGGCGCAACTAATTTTAGTCTACTGAAACAAGCTGGTGATGTGGAGGAAAATCCAGGACCAatggtagcaggtcatgcctct
74	miRFP670_rev	ctgtacaagtaattagctctcaagcgcggtgat
75	cmv_seq_for	tccgGCCACCATGGGat
76	p2a_seq_for	GGTGATGTGGAGGAAAATCCAGGACCAa
77	cib81_for	ACGACGGTCTCTCATCGAATGGAGCCATCGGCG
78	cib81_rev	agtaggtctctacgcagcgcttcccGAGATAGACAACCTAGAATCTCC
79	PDZ_for	ACGACGGTCTCTCATCGCAAAGGAGGCGAGTGACTG
80	PDZ_rev	agtaggtctctacgcagcgcttcccCATATATTTCACTTCCAATACTACTTCCT
81	med_flexi_for	GAGACCctgaGATCGgcgc
82	med_flexi_rev	cgatgccaactcgtcaacc
83	long_flexi_for	AGCACGGGACTGTTACGAGC
84	long_flexi_rev	cctgtcGATCGGATCGTcgc
85	cib81_gs3_for	ACGACGGTCTCTCATCGGGATCAGGTAATGGAGCCATCGGCGG
86	cib81_gs3_rev	agtaggtctctacgcACCGCTTCCCGAGATAGACAACCTAGAATCTCCTT
87	cib81_gs5_for	ACGACGGTCTCTCATCGggctccGGATCAGGTAATGGAGCCATC
88	cib81_gs5_rev	agtaggtctctacgctccggaACCGCTTCCCGAGATAGA
89	cib81_gs9_for	ACGACGGTCTCTCATCGggttccggatcgggctccGGATCAGGTAATGGAGCCATC
90	cib81_gs9_rev	agtaggtctctacgcgcctgaaccgcttccggaACCGCTTCCCGAGATAGA
91	cry2_for	agcagCGTCTCtcagatccgatgAAAATGGATAAGAAAACCATAGTGTGGTTCAG
92	cry2_rev	agacagCGTCTCCAGCTTGTTTCAGTAGACTAAAATTAGTTGCGATCCCAATGCGACCGC
93	mkate_for	agacCGTCTCAAGCTGGTGATGTGGAGGAAAATCCAGGACCAATGAGCGAGCTGATCAAAGAAAA
94	mkate_rev	agacaCGTCTCaagtttTTATCTGTGGCCCAGCTTAGA
95	cry2_mkate_backbone.for	agacaCGTCTCgaacttgtttattgcagcttataatggttacaaataaagc
96	cry2_mkate_backbone.rev	agacaCGTCTCatctgacggttcactaaaccagct
