# Supplementary material for: Domain insertion permissibility-guided engineering of allostery in ion channels
Source: Nat Commun. 2019 Jan 17;10:290. doi: 10.1038/s41467-018-08171-0 (PMC6336875; doi:10.1038/s41467-018-08171-0)
Supplement: Supplementary file 2 — Supplementary Info File—reporting summary [file 41467_2018_8171_MOESM2_ESM.pdf]

nature research

Corresponding author(s): Daniel Schmidt

Reporting Summary

Nature Research wishes to improve the reproducibility of the work that we publish. This form provides structure for consistency and transparency in reporting. For further information on Nature Research policies, see Authors & Referees and the Editorial Policy Checklist.

Statistical parameters

When statistical analyses are reported, confirm that the following items are present in the relevant location (e.g. figure legend, table legend, main text, or Methods section).

n/a	Confirmed	
☐	☒	The exact sample size (*n*) for each experimental group/condition, given as a discrete number and unit of measurement
☐	☒	An indication of whether measurements were taken from distinct samples or whether the same sample was measured repeatedly
☐	☒	The statistical test(s) used AND whether they are one- or two-sided<br>*Only common tests should be described solely by name; describe more complex techniques in the Methods section.*
☒	☐	A description of all covariates tested
☐	☒	A description of any assumptions or corrections, such as tests of normality and adjustment for multiple comparisons
☐	☒	A full description of the statistics including central tendency (e.g. means) or other basic estimates (e.g. regression coefficient) AND variation (e.g. standard deviation) or associated estimates of uncertainty (e.g. confidence intervals)
☐	☒	For null hypothesis testing, the test statistic (e.g. *F*, *t*, *r*) with confidence intervals, effect sizes, degrees of freedom and *P* value noted<br>*Give P values as exact values whenever suitable.*
☒	☐	For Bayesian analysis, information on the choice of priors and Markov chain Monte Carlo settings
☒	☐	For hierarchical and complex designs, identification of the appropriate level for tests and full reporting of outcomes
☒	☐	Estimates of effect sizes (e.g. Cohen's *d*, Pearson's *r*), indicating how they were calculated
☐	☒	Clearly defined error bars<br>*State explicitly what error bars represent (e.g. SD, SE, CI)*

Our web collection on statistics for biologists may be useful.

Software and code

Policy information about availability of computer code

Data collection	Flow data was collected using FACSDiva v.8.0.1 and electrophysiology data was collected using SutterPatch (v2.0.0).
Data analysis	Domain insertion permissibility alignment and enrichment was done using the published Dip-Seq pipeline developed by David Savage and coworkers, which were only slightly modified for compatibility with updated python packages. Permissibility was mapped onto structures using USF Chimera, v 1.11.12 (build 41376). Further numerical data analysis (correlation, decision tree models, etc.) was done using R version 3.5.1 (2018-07-02). were updated regularly and the most recent version was used. Version number for specifically mentioned packages are: rpart (4.1-13), flowStats (3.36.0). Flow cytometry analysis was done in FlowJo 10. URLs for webservers used in computing properties (e.g., EVmutation, iGMN2.0, SWIFT) are cited. Many to one comparison were done use the PMCMRplus package (version 1.4.0). Electrophysiology data was exported from SutterPatch v2.0.0 before being further analyzed in R.

For manuscripts utilizing custom algorithms or software that are central to the research but not yet described in published literature, software must be made available to editors/reviewers upon request. We strongly encourage code deposition in a community repository (e.g. GitHub). See the Nature Research guidelines for submitting code & software for further information.

Data

Policy information about availability of data

All manuscripts must include a data availability statement. This statement should provide the following information, where applicable:

- Accession codes, unique identifiers, or web links for publicly available datasets
- A list of figures that have associated raw data
- A description of any restrictions on data availability

The datasets generated during and/or analyzed during the current study are available from the corresponding author on reasonable request. The source data underlying Figs. 2-4, 6a, 7b and Supplementary Figs. 3-10 is provided as a Source Data file in the Sequence Raw Archive (SRA – https://www.ncbi.nlm.nih.gov/sra) accession code for the data is PRJNA506141 [https://www.ncbi.nlm.nih.gov/bioproject/?term=PRJNA506141].

Field-specific reporting

Please select the best fit for your research. If you are not sure, read the appropriate sections before making your selection.

☒ Life sciences ☐ Behavioural & social sciences ☐ Ecological, evolutionary & environmental sciences

For a reference copy of the document with all sections, see nature.com/authors/policies/ReportingSummary-flat.pdf

Life sciences study design

All studies must disclose on these points even when the disclosure is negative.

Sample size	No sample size calculation was performed. All raw data collections were performed with a least three biological / independent replicates which is a commonly accepted standard.
Data exclusions	One sample NextGen sequencing sample had too few reads for either 'GFP' and 'Label' in Cib81 and PDZ datasets and was therefore excluded. The three biological replicate threshold was still met. Whole cell recordings with access resistance > 40Megaohm were excluded. Unstable cell-attached recordings that developed excessive leak over the course of the experiment (baseline current drift by >10pA) were excluded.
Replication	All raw data collections were performed with a least three biological / independent replicates that confirmed reproducibility.
Randomization	Randomization was not relevant to this study as samples are not divided into experimental groups.
Blinding	Experimenters were not blinded; it seemed not necessary. As this was a discovery-driven project, and in the absence of a theory to be disproven, there wasn't any bias one way or another. For light-switching experiments, different experimentalists conducted flow cytometry and electrophysiology experiments, which bore out the same conclusions.

Reporting for specific materials, systems and methods

Materials & experimental systems

n/a	Involved in the study	
☒	☐	Unique biological materials
☐	☒	Antibodies
☐	☒	Eukaryotic cell lines
☒	☐	Palaeontology
☒	☐	Animals and other organisms
☒	☐	Human research participants

Methods

n/a	Involved in the study	
☒	☐	ChIP-seq
☐	☒	Flow cytometry
☒	☐	MRI-based neuroimaging

Antibodies

Antibodies used	anti-FLAG M2 monoclonal (Sigma Aldrich)
Validation	Validation is based on manufacturers lot documentation and certificate of analysis. Further validation are positive and negative controls included in the experimental design.

Eukaryotic cell lines

Policy information about cell lines

Cell line source(s)	HEK293FT (Thermo Fisher Scientific)
Authentication	Not authenticated.
Mycoplasma contamination	Not tested for mycoplasma.
Commonly misidentified lines (See ICLAC register)	Cells claimed to be HEK may be in fact HeLa. HEK293FT are standard cell line used by hundreds of labs around the world. Thermo Fisher Scientific is a reputable vendor that verifies cell line identity by Tandem Repeat Analysis.

Flow Cytometry

Plots

Confirm that:

☒ The axis labels state the marker and fluorochrome used (e.g. CD4-FITC).

☒ The axis scales are clearly visible. Include numbers along axes only for bottom left plot of group (a 'group' is an analysis of identical markers).

☒ All plots are contour plots with outliers or pseudocolor plots.

☒ A numerical value for number of cells or percentage (with statistics) is provided.

Methodology

Sample preparation	Sample preparation for permissibility assays is described in the Methods (Domain insertion permissibility cell sorting assay). Sample preparation for function assays is described in the Methods (Resting membrane potential functional assay). Sample preparation is described in the Methods (Flow Cytometry Assay for Light-modulation of Kir2.1 function).
Instrument	BD FACSAria II P69500132 and BD Fortessa H0081 flow cytometer
Software	FACSDIVA v8.0.1
Cell population abundance	For permissibility assays, between 2,000-100,000 cells were collected for each sample/library pair. For function and light-switching assay >200,000 cells were analyzed.
Gating strategy	Gating strategies for permissibility assays, function assay, and light-switching assay are described in the Methods and Supplementary Figures 12-14.

☒ Tick this box to confirm that a figure exemplifying the gating strategy is provided in the Supplementary Information.
